# Clinical Review, and Hospital Reports

**Published:** 1842-01-01

**Authors:** 


### Clinical Review.

### GUY'S HOSPITAL.

Guy's Hospital Reports. No. XIII. October, 1841. Edited by George H. Barlow, M.A. & M.D. Trinity College, Cambridge; and James P. Babington, M.A. Trinity College, Cambridge.

The present Number contains fewer papers than some of its predecessors have done. The Contents are as follows :—

Observations on certain Diseases originating in Early Youth; illustrated by Cases of defective Expansion of the Lungs; by George H. Barlow, M.A. & M.D. :—Medico-Legal Report of the Evidence given on a recent Trial for Murder by Poisoning with Arsenic; by Alfred S. Taylor :—A remarkable Case of Abdominal Effusion, resulting from Mesenteric Tumor; by H. Marshall Hughes, M.D. With Observations on the Effused Fluid, by G. Owen Rees, M.D. :—On Acute Aortitis: a Portion of an Essay read before the Physical Society at Guy's Hospital, Jan. 13, 1838; by Norman Chevers, M.D. :—Cases of Malignant Disease of the Lung; by H. Marshall Hughes, M.D. :—Reports of Cases requiring Capital Operations, which have been performed since Oct. 1, 1840, by Bransby B. Cooper, Esq. F.R.S. :—On the Structure of the Blood Corpuscle; by G. Owen Rees, M.D. and by Samuel Lane :—Account of a Patella broken transversely, and re-united by Bone. With Remarks on the Nature and Treatment of similar Injuries; by T. Wilkinson King. (With Plate) :—A Case of Intestino-Vesical Fistula; by Mr. Hingeston. Communicated by Mr. C. Aston Key. (With Plates) :—On Chorea; by B. G. Babington, M.D. F.R.S.

#### I. Observations on certain Diseases originating in Early Youth; illustrated by Cases of defective Expansion of the Lungs. By George H. Barlow, M.D.

Dr. Barlow remarks :—

"There is, I think, one consideration connected with the ætiology and treatment of a large class of chronic and organic diseases, which, although it cannot have escaped the notice of any practitioner of experience and observation, does not appear to have received that attention which its importance seems to demand;—I mean, the occurrence of disease arising from a want of proportional development in some of the organs of the body; by which, either, other organs are excited to undue activity, leading to their excessive development and ultimate disorganization; or, serious, and sometimes fatal, disturbances are produced in the current of the circulation.

The deviation from the normal proportion, to which I more particularly allude, exists in the lungs, the liver, and the two sides of the heart; and its first commencement, or, at all events, its first manifestations, may generally be traced to the period of early youth; when, owing to the increasing proportion of those parts whose blood is returned to the heart directly by the venæ cavæ, to those which return it through the medium of the portal system, a corresponding change takes place in the relative volume and functions of the liver and the lungs; the latter being developed, as it were, at the expense of the former. When the lungs and heart are sound, the air-passages unobstructed, and the capacity of the chest sufficiently ample, this change is effected with safety; and that full expansion of the chest is the result, which the artist, as well as the physiologist, has always regarded as indicative of health and vigour. But if, on the other hand, the expansion of the lungs be impeded, either by a want of capacity in the chest, or narrowing or obstruction of the principal air-passages—or if the lungs be unsound—or if the circulation be disturbed by disease in the heart, or a want of due proportion between its different cavities and its different orifices—or if the extremities be too rapidly developed—the consequence must be, either, that the change which should now take place is prevented, and the liver retains its original proportion to the rest of the body, the result being a tumid abdomen and contracted chest, with imperfect respiration, and stunted and often œdematous extremities—or, that the lungs suffer, either from undue accumulation of blood, or from the excess of function which they may be called upon to perform—or the heart becomes embarrassed, and ultimately diseased, by obstruction to the current of the circulation."

Dr. Barlow, however, confines his present paper to those cases in which the expansion of the lungs is impeded either by contraction of the chest or defect in the principal air-passages.

The first case is given at great length. Its nature may be guessed from the following summary of its particulars—dyspnœa first becoming urgent about the age of 11—palpitation, ascites, and anasarca—checked growth—partial recovery—occasional relapses—severe return of symptoms at the age of 15—no signs of puberty—death at the age of 16—distention, and hypertrophy of the right side of the heart.

In some observations on the case, which is much too long for insertion, Dr. Barlow develops his ideas upon its nature. He says that the prominence of the heart symptoms during life and its enlargement discovered after death, must not be regarded as the source of the mischief.

"It appears," he remarks, "that up to the age of 10 or 11 years, this patient had enjoyed good health, so that there could not have existed any abnormal condition of the heart before that time; whereas the healthy state of the valves, and the absence of all traces of any inflammatory affection of either surface of the heart, leave us without any assignable cause, referrible to that organ, for the hypertrophy which was afterwards found to exist; the change being, in fact, just such as might be expected to result from obstruction to the passage of the blood from the heart. And further, the hypertrophy and distention existing in so much greater a degree on the right side, shews that this obstruction must have existed, for the most part, between the right and left heart; namely, in the pulmonary circulation. If, moreover, we refer to the history of the case, we shall find abundant evidence of the existence of such obstruction, in the dyspnœa, the lividity of the countenance, the narrowness of the chest, and the dropsical effusion observed during life; as well as in the compressed state of the lungs, the congestion of the liver, the narrowness of the pulmonary artery, and the enormous distention of the right auricle and ventricle, which were found after death.

But, although the obstruction to the passage of the blood through the lungs must act immediately, and therefore with the greatest effect, upon the right side of the heart, yet the obstruction thus afforded to the return of the blood through the systematic and portal veins must increase the difficulty with which the blood passes into the veins from the arteries, and in this manner call upon the left ventricle for an additional effort; and consequently give rise to distention there, though in a much less degree than on the right side of the heart. It appears, then, that we have a cause adequate to the production of the abnormal state of the heart; and, moreover, have satisfactory proof of the existence of that cause."

We find in the account of the dissection of the case, that the left auricle was dilated and hypertrophied, but in a much less degree than the right. When the auricle was laid open, the auriculo-ventricular opening was seen, of the size of a shilling. The curtains of the mitral valve were very slightly thickened, and contracted. The cavity of the left ventricle was large, and its walls rather thickened.

It appears to us, if we rightly apprehend Dr. Barlow's statement, that there was narrowing of the left auriculo-ventricular opening. If this were so, it would go to explain much of the case and rather militate against Dr. Barlow's observations.

The SECOND CASE was one of *Dyspnœa first becoming urgent about the age of* 15—*Bronchitis—Lividity—Palpitation—Severe aggravation at the age of* 18 —*Anasarca—Ascites—No signs of puberty—Death.*—Like the former, it is reported too much at length for insertion. But the observations of Dr. Barlow upon it will convey his ideas to our readers.

"In this case, as in the last, there can, I think, be little difficulty in accounting for the more urgent symptoms, by the obstruction to the return of the venous blood; of which sufficient evidence is afforded by the distended state of the right auricle.

The action of this obstruction, in producing venous congestion as well in the system generally as in the portal circulation, and thereby giving rise to lividity, anasarca, and ascites, is too obvious, and too generally acknowledged, to be here insisted upon. The next point, then, is the cause of the distention found to exist in the right auricle. This was obviously the effect of the opposition offered to the passage of the blood from the auricle to the ventricle, and to the regurgitation which no doubt took place through the tricuspid valve: and here also, as in the former case, the condition of the right ventricle is to be accounted for by the distention arising from the obstruction to the passage of the blood from the right side of the heart; for which we have a cause, in the smallness and diseased condition of the pulmonary arteries. Was then the defect, primarily, in the pulmonary arteries? or must we still seek for some anterior cause? Now, upon a reference to the first case, we find a small trachea and bronchi; small chest; tough and fleshy lungs; with enormous dilatation of the right ventricle and auricle, and patulous tricuspid valve: in the second, we have a similar state of things, as regards the lungs, and walls of the chest; with the addition of the disease of the pulmonary artery, and the dilatation of the bronchial tubes: the condition of the walls of the right side of the heart was nearly the same in both cases, though the dilatation and hypertrophy existed in a greater degree in the first than in the second. On the left side there was considerable disease of the mitral valve, in the second case, though but little, if any, in the first. The identity of the lesion, therefore, as regards the right heart, (the difference in the two cases being only in degree,) naturally suggests the idea of an identity of cause: and in seeking for that cause, we are, I think, justified in rejecting as adventitious what was found only in the second case, namely, the disease in the coats of the pulmonary arteries and mitral valve; and retaining, as essential, what was common to both, namely, the obstruction offered by the compressed state of the lungs, and narrowness of the pulmonary arteries.

The next question which arises is: are these conditions of the lungs and pulmonary artery to be regarded in the light of cause and effect; or as the collateral effects of some anterior cause, to be sought for hereafter? Now, reasoning from analogy, we should, I think, be led to the conclusion, that the diminished calibre of the artery was the result of the diminished capacity for blood of the part supplied by that artery, as is seen in the case of the contraction of an artery above a ligature; in the variation in size of the spermatic and uterine vessels; and in other instances, which it would be superfluous to enumerate.

We come next to the inquiry, Whether the ill-developed condition of the lungs was the primary disease? or, Whether either of the two other circumstances observed in both cases, namely, the diminished capacity of the chest, and the diminished calibre of the trachea, should be regarded as an anterior cause ? Now, as regards the first, it is, I think, agreeable to the analogies which might be drawn from other parts of the body, to suppose that the softer and more important part gives form to the harder and less important, rather than the harder to the softer; as the scull is indented by the convolutions and vessels of the brain; and bone is absorbed before the pressure of an aneurismal tumor. It is therefore more probable, that the narrowness of the thoracic walls was the effect of the diminished bulk of the lungs, than that the contrary was the case.

Again: Are we to regard the smallness of the trachea as cause, or effect, of the diminished capacity of the lung ? It must be confessed, that, anteriorly, we should be as much inclined to regard the size of the duct as dependent upon that of the gland, as that of the gland upon the calibre of the duct. But of this, direct proof is wanting; whereas it is ascertained—as in the case quoted by Dr. Stokes, from Reynaud—that obstruction to the air-passages causes atrophy of the lungs. We may therefore regard the narrowing of the trachea as a *vera causa*, and one capable of accounting for the phænomenon for which we are seeking a cause."

Now we are told in the dissection, that "the left auricle was considerably dilated and thickened: the mitral valve was occupied by a mass of ossified matter, partially denuded; and the curtains contracted towards each other, so as to diminish the auriculo-ventricular opening to a circular aperture of flexible but thickened membrane, which would not admit more than the end of the little finger." We may be in error, but we would certainly be inclined to attach much more consequence to this lesion than Dr. Barlow seems to do. Here is a positive obstruction—its effects (dilatation of the left auricle and right-heart, and pulmonary apoplexy) palpable. Why should it be put in a parenthesis and more hypothetical and questionable alterations paraded in the foreground ? We have seen the very same changes, the very same symptoms in adults of such conformation of thorax as to preclude the notion of defect in that quarter.

And, by the way, if, in the first case, the obstruction lay in the lungs, why should the *left* auricle have been dilated and hypertrophied. It *ought* to have been the contrary. It is only fair to hear Dr. Barlow on the other side.

"There are two other remarkable lesions to be noticed, which were observed in the second case, but not in the first; namely, the disease of the mitral valve, and the pulmonary apoplexies. Of these, the latter, which were recent, were, in all probability, the effect of the former, which was evidently of long standing. The manner in which these must have been brought about, need hardly be pointed out: for the powerful right ventricle must, when the return of the blood from the lungs was opposed by the diseased condition of the auriculo-ventricular opening, have forcibly compressed the pulmonary tissue: and we cannot wonder that extravasation was the result. The disease of the mitral valve, however, deserves consideration upon another account; for it may be said, that it, and not the smallness of the trachea, was the cause of the non-expansion of the lung, by the impediment which it offered to the pulmonary circulation. To this there appears to me to be the objection, that in the first case, where the pulmonary obstruction existed in an equal degree, there was no disease of the left auriculo-ventricular opening. And secondly, that in those cases where there has existed engorgement of the right heart, either from disease in the left auriculo-ventricular opening, or from compression of the lungs by the narrowing of the thoracic walls, we do not have diminished calibre of the air-passages. Neither is the same effect produced on young persons, in whom the capacity for air in the lungs is diminished by the presence of tubercles in those viscera. Unless, then, we adopt the hypothesis of the narrowing of the trachea being the principal source of the morbid phenomena, in this, as well as in the first case, we are compelled to regard it as a mere accidental coincidence, without cause, and without effect."

We confess that we are not convinced, and that more evidence is required to satisfy us of the operation of this cause. Dr. Barlow, however, reasons his case bravely, as the following ingenious passage will prove.

"This brings me to the left ventricle; which was hypertrophied, as well as dilated, in both cases. In the second, there was perhaps a twofold cause for this; namely, the venous congestion, and the disease in the coats of the aorta. In the first case, however, the aorta and its branches were healthy, and therefore the effect must be ascribed wholly to the venous obstruction. Of the action of this obstruction upon the left ventricle, I have before spoken, and shall have occasion to notice it more at length hereafter; and am only induced to recur to it now, as I believe that a different view is taken, by many, of the effect of pulmonary obstruction upon the left side of the heart. In the case of Mary K——, the diminution of the pulmonary artery, as well as the reduced (though otherwise healthy) left auriculo-ventricular opening, shew that there must have been a diminution in the current which flows from the right to the left side of the heart. This diminution must have acted as a relief to the left ventricle; and so far would have produced the opposite effect to dilatation and hypertrophy. On the other hand, the obstacle opposed to the passage onward of blood in the veins, and the ultimately indurated condition of several organs through which a circulation was to be maintained by the left ventricle, must have tended to embarrass that ventricle, and therefore to produce dilatation, or dilatation with hypertrophy: so that there were two opposite causes in operation; and, accordingly as the former or the latter might preponderate, we should have defect or excess in the force distending the left ventricle. Now, in the case before us, we should have expected, *à priori,* that the orifices leading to and from the ventricle would have adapted themselves to the quantity of blood which they had to transmit, and such would also have been the case with the arteries: and it is probable, that, at an early period, the whole systemic circulatory apparatus (the arteries as well as the ventricle) was small and unembarrassed. But when the increasing distention on the right side gave rise to regurgitation into the venous trunks, and ultimately to congestion in the smaller veins, and induration of the liver, and perhaps also of the cellular membrane of the extremities, there would be a fresh obstacle to the flow of blood in the arteries—that is, a fresh obstacle to be surmounted by the efforts of the left ventricle; which would, if the powers of the system were not materially impaired, lead to dilatation with hypertrophy. We should, then, upon this hypothesis, expect a large and strong left ventricle with small auriculo-ventricular opening, and small aorta; and such were actually found to exist. It is not necessary, then, that obstruction to the pulmonary circulation, especially in young persons, should give rise to atrophy of the left ventricle."

And now for the moral.

Dr. Barlow divides his therapeutic measures into the preventive, the remedial, and the palliative.

1. The *preventive* means, he thinks, will be most readily suggested by a review of the origin and progress of the disease. Thus a young person about eleven or twelve years old is observed to be short breathed, perhaps to be troubled with a cough and to be rather bloated in the face, and is, perhaps, sent home from school on that account: measures, in themselves judicious, are very likely adopted; and considerable relief is obtained. The child, perhaps, returns to school; and the mother and governess, or tutor, and very likely the medical man too, comfort themselves in the belief, that, if a boy, he will "out-grow it"; and if a girl, that, when "a certain change" takes place, all will be right: but it somehow happens, especially if the patient be placed under unfavourable circumstances, or if the original disproportion be great, the boy, instead of "out-growing" his complaint, ceases to grow at all; or the expected "change" in the girl never takes place; and hypertrophy and dilatation of the right heart, with the train of symptoms detailed in the case of Mary K——, are the consequence: or perhaps the boy does in some measure out-grow his complaint, or for a time appear to have done so; and the girl attains to womanhood; but cough and dyspnœa, with perhaps asthmatic paroxysms, occur, at first, most probably after considerable intervals; but afterwards more frequently, and for a longer continuance; and ultimately become permanent, and carry off the patient at a premature age, or render his life a state of protracted helplessness and suffering.

The period for preventive measures is early youth. The most appropriate appear to be, to endeavour to develop the respiratory apparatus, to maintain a regular and moderate action of the other excretory organs; and to obviate, as much as possible, all venous congestion, by regulating the amount of the circulating fluid.

For fulfilling the first of these indications, a pure air is absolutely essential. An atmosphere loaded with carbonaceous vapours, or the exhalations from decomposing organic matter, not only, by its disagreeable qualities, tends to repress the healthy action of the lungs, but also acts, as has been already observed, as a direct stimulus to the liver: whereas a pure atmosphere, free from such noxious matters, and containing the due proportion of oxygen, is the natural stimulus of the respiratory organs, and must therefore tend to promote their healthy development.

Again: the regulated exercise of the system generally, as well as of the lungs in particular, must not be overlooked. The former not only, by exciting, gives tone and vigour to the vascular as well as the muscular system, but also, by increasing the rapidity of the venous current, calls the lungs into fuller action. It is almost needless to observe, that if such exercise be carried to excess, the effect will be one of the very evils which we are desirous of preventing; namely, the engorgement of the right heart: and therefore it is necessary, that where there exists any tendency to the affection of which we are treating, all such exercise as produces palpitation of the heart, or any tendency to lividity of the countenance, should be carefully restrained. The inclinations of the patient, unless he be stimulated by emulation to perform some feat of activity or muscular strength, will generally be a sufficient safeguard. The exercise here recommended should also be taken as much as possible in the open air.

The advantage of exercising the lungs themselves is not perhaps, in general, sufficiently considered: yet no one can contemplate a child shouting in the joyousness of his heart, without believing that he is obeying an instinct given by nature for some useful purpose. This instinct in children seldom, indeed, needs to be encouraged; though there is reason to apprehend that it is often mischievously and cruelly repressed. The advantage of moderate exercise of the vocal organs is equally great in early youth as in childhood; and perhaps the singing lessons are of some service in this respect, though they do not generally commence at the time when the expansion of the lungs is of the most importance. Reading aloud is another means of expanding the lungs and air-passages; which is most beneficial, unless carried to excess:—and thus the acquisition of the most useful and agreeable of accomplishments may be made subservient to health. Upon the importance of a careful attention to the state of the excretory organs in childhood and early youth, it is hardly necessary to insist: at the same time, it may be well to recommend caution in effecting our purpose; for it must be remembered, that over-stimulating leads often to disorganization. When the evacuations appear to be deficient in bile, or there is any reason to apprehend congestion in the portal system, a mercurial purgative will be most beneficial; but it should never be wantonly administered. A free and regular action of the kidneys should also be maintained: where they appear to act sufficiently, the use of diuretics should be avoided; but if, on the contrary, there appears to be any deficiency in the quantity of the secretion, the least stimulating means for the encouraging it should always be preferred. Dr. B. recommends "Imperial"—acetate of potass—digitalis; warm clothing to regulate the functions of the skin; moderation in the taking of fluids.

Dr. Barlow next speaks of the remedial means, those calculated to restrain the increase of the disproportion when it already exists; and thus to allow an opportunity for the healthy relation of the several organs to be restored, before the period when growth ordinarily ceases. Dr. B. admits the difficulty of diagnosis. He believes, however, that the first indication is to relieve the lungs by exciting to greater activity the liver, kidneys, and skin. The next object must be, to control the action of the heart, and to prevent its embarrassment, either from a too great quantity of blood in the system generally, or its accumulation in the venous trunks; and, lastly, to favour the development of the defective organs, by such measures as have been already suggested. The means of fulfilling these indications must, for the most part, be left to the discretion of the practitioner. Dr. B. speaks highly of the combination of calomel, digitalis, camphor, and hyoscyamus. Moderate abstraction of blood will also be occasionally beneficial.

We must also bear in mind the liability of the patient to pulmonary apoplexy from any cause which may determine an increased quantity of blood to the right heart; and also to lesion of the tunics of the arteries, from any unusual excitement of the heart's action. The pernicious practice of compressing the lower parts of the lungs, by tight stays or other parts of dress, cannot be too strictly forbidden.

As for the palliative treatment, the cautious abstraction of blood will sometimes be found beneficial; and the careful use of digitalis will be found serviceable, in controlling the heart's action. The great object, also, of relieving the lungs by means of the auxiliary secretory organs should never be lost sight of: for this purpose, the bowels should be freely acted upon, and gentle diaphoresis maintained: and if free diuresis can be produced, we shall effect the purpose of carrying off redundant fluid, and thereby relieving the heart, whilst we are fulfilling the important indication just pointed out.

### II. Medico-legal Report of the Evidence given on a recent Trial for Murder by poisoning with Arsenic. By Alfred S. Taylor.

This is a *very* valuable report. But the case on which it is founded, as well as the Report itself, are too long and too minute for insertion in these pages. All that we can do, is to pick out some observations or some hints that may be useful.

1. *Inadequacy of common Reports.*—The slow progress of toxicology in England must be ascribed to the want of a sufficient number of accurate reports. A medical witness has generally gone into court, to give evidence in a case of poisoning, without any knowledge of the kind of information which has been required by counsel, from those who have acted as witnesses in former cases. The report of a trial, if published at all, has commonly appeared, either in an incorrect form in the public journals, or divested of all that is useful to the medical witness, in various works on criminal law.

2. *What a Witness may be asked.*—Each trial affords a lesson: it at least teaches us what we are expected to know, and what we must be prepared to answer. At first, the view of the counsel for the defence seemed to be, that it was possible the diarrhœa, which the deceased was known to have had two days before his death, might have carried him off: but, independently of this not being a fatal disorder in an otherwise healthy man, the medical opinion was conclusive, that the deceased must have died from poison. Mr. Breach was then asked, whether arsenic might not have been accidentally mixed up in the powders sent by him to the deceased, and whether he kept arsenic among his medicines. The answers were, That he prepared the powders himself—that he had prescribed similar powders on the same day for numerous other persons, without any ill effects resulting—and that the only arsenical preparation which he had in his possession was in a state of solution. He was required to describe the method of analysis adopted for the detection of the poison;—to account for the safe custody of the stomach and its contents, from the time of removing them from the body until the analysis was made, as also for the safe custody of the tubes and plates containing the arsenic reduced to the metallic state. The counsel then required him to describe the probable quantity of reduced metal obtained in each tube; and to say whether it would be possible to convert all the poison, diffused through the stomach and intestines, to the state of metal.

The questions put to myself, in cross-examination, were chiefly—Whether ulceration of the mucous membrane of the stomach was a common effect of arsenical poisoning—What was the earliest period, after the poison had been taken, at which ulceration might take place—the earliest period for the occurrence of severe inflammation of the mucous membrane,—the quantity of arsenic required to kill an adult—how much was found in the deceased's stomach, and whether this was sufficient to destroy life. Again: How many tests were employed for the detection of the poison—which was the most satisfactory of these—and why, if the test of reduction was satisfactory, were others afterwards employed.

3. *Earliest Period for Ulceration of the Stomach from Arsenic.*—" The mucous membrane of the stomach presented patches of *ulceration*. The earliest period at which I have known this process to have been set up, in arsenical poisoning, is seventeen hours. Dr. Christison remarks, that it is hardly to be looked for, unless the patient has survived nearly two days. In the case before us, the deceased died within *ten* hours, and his stomach was found ulcerated. Both Mr. Breach and myself were closely cross-examined by prisoner's counsel on this point; the object being probably to shew, that, according to all past experience, it was too early for the ulceration met with to have been the result of arsenic. But the appearance of the ulcers, with the fact of the poison being embedded in them, left no doubt of their origin on our minds, and so we did not hesitate to express ourselves. It was further explained, that as ulceration was a consequence of the actively inflamed state of the mucous membrane from the local irritation produced by arsenic, so it might happen that this appearance would be accelerated where the inflammation had been unusually acute."

4. *Is direct Evidence of Guilt necessary?*—The cases of poisoning in which direct evidence of administration can be procured, are, comparatively, very few in number. Poisoning is a crime almost always perpetrated in secresy: the murderer, in general, so lays his plans, that there may be no witness to the act of administration. These plans sometimes fail; but when they succeed, there are often circumstances which betray the nature of the crime, as well as the perpetrator. It is upon these circumstances, partly moral and partly medical, that courts of law commonly rely for proof of administration; and there can be no doubt, that if direct proof were always required for conviction, it would be tantamount to saying, that the greater number of cases of criminal poisoning are entirely beyond the reach of the law; and that the crime might be perpetrated with impunity, provided a few very simple cautions were observed by the perpetrator.

### III. A Remarkable Case of Abdominal Effusion, resulting from Mesenteric Tumor. By H. Marshall Hughes, M.D. With Observations on the Effused Fluid. By G. Owen Rees, M.D.

This case occurred in the person of a young man, a patient of Dr. Hughes, at the Surrey Dispensary. He had symptoms of dyspepsia, followed by effusion into the abdomen, which quickly increased with remarkably rapid emaciation. In less than two months from his first application he died.

*Dissection.*—The peritoneal cavity contained from seven to eight quarts of rather thick and perfectly milky fluid, aptly compared, from its appearance, to almond emulsion. The peritoneum was not vascular, excepting over a depending portion of the ileum, but was universally sprinkled with minute white specks; by far the larger portion of which was easily removed by gentle friction, and consisted of delicate and almost capillary shreds, evidently deposited from the milky fluid; but some of which were as clearly firmly adherent to, if not produced by, the membrane itself. They were translucent, angular, and elongated, rather than rounded; and, in external appearance, bore a much more striking resemblance to the ova of *pediculi capitis* than to any form of tubercles. In the centre of the abdomen, resting on the spine, was a rounded nodulated tumor, as large as a twopenny loaf, which consisted of several agglomerated mesenteric glands; some of which were as large as a small orange, and, when divided, presented a soft, pinkish, pultaceous mass; from which, upon very slight pressure, exuded a white cream-like fluid, which appeared to constitute a portion of the deposit itself: others were of a dull white colour, drier and more granular; the whole exhibiting, both exteriorly and upon the exposed section, the general characters of cerebriform cancer. Other glands of the mesentery were more or less enlarged and opaque, some equalling the size of marbles and of pigeons' eggs. Some of the inguinal glands were also considerably enlarged, but contained no heterologue deposit. Several convolutions of the intestines, and the transverse arch of the colon, were adherent to the tumor. They all, however, appeared healthy, except the colon, which, in two places, was contracted and puckered around two white spots as large as a shilling, which were white, firm, and semi-cartilaginous in appearance. Opposite to these spots, the mucous membrane was entirely wanting; and their cut surface presented the same physical characters as the early stages of schirrous pylorus. One tubercular-looking body *only*, about the size of a pea, was discovered in the mesentery, close to a fold of the ileum. The liver, spleen, and kidneys were healthy. The pancreas was, unfortunately, not examined. Numerous lacteals—large tortuous, varicose, and distended, some with a milky, and others with a clear fluid—were observed in almost all parts of the mesentery: but in consequence of the sections already made, and of the examination occurring in a private house, no attempt at injection, for the purpose of discovering any lesion, could be made with any probable chance of success. Six ounces of the fluid were transmitted to Dr. Rees, for examination, who replied:—

"I have examined the effused fluid, and find that it contains chyle in considerable quantity. Owing to the chemical character of serous effusions generally, it is quite impossible to determine what quantity of chyle is in admixture with the serum. Some idea, however, may be formed of its large proportion, when we recollect the peculiar milky appearance (so distinct from that of pus), which seems immediately to have attracted your attention. When this effusion was agitated with ether, it separated into three distinct parts; the upper being a solution of fatty matter in ether, the lower a clear serum, and the intermediate layer a floating mass of chylous matter.

This chylous matter, to which I have drawn attention in a late number of the Medical Gazette, is analogous to an animal principle existing in large proportion in the saliva, and which seems to play some very important part in the process of nutrition. The characters of the fatty matter dissolved by ether, considered in connexion with the separation of the peculiar principle above alluded to, and the clearing of the serum consequent on the action of ether, render it pretty certain that the milkiness was owing to the presence of chyle. I certainly have never seen, and do not recollect ever to have heard, of an effusion presenting the chemical characters observed in this specimen."

The symptoms, in this case, depended, no doubt, on the pressure of the tumor. This was probably malignant. The tumor pressed on the branches contributing to the vena portæ and the thoracic duct: the passage of the chyle through some of the mesenteric glands was, in all probability, nearly, or perhaps entirely, prevented by the disease affecting their structure: hence arose, not only the serous effusion, and the repletion and tortuosity of, but exudation from, or actual rupture of, the chyliferous ducts: hence also, though the appetite was not very deficient, the wasting of the body was most extraordinary both in degree and celerity; and hence the existence of chyle in the fluid. Such is Dr. Hughes's rationale of the case.

### IV. On Acute Aortitis: a portion of an Essay read before the Physical Society of Guy's Hospital, January 13, 1838. By Norman Chevers, M.D.

Dr. Chevers observes that acute inflammation of the thoracic aorta appears to occur, in nearly all cases, either as a direct result, or as a complication of some other morbid process co-existing in the system. Thus, for example, it sometimes happens that acute inflammation, originating in the endocardium, extends onwards upon the membrane of the aorta. Again, in instances of pericarditis, the great artery has been known to participate in the disease; but this state has generally been noticed in conjunction with very extensive inflammation of the other thoracic serous membranes. In some extreme cases of pleurisy and pneumonia, occurring in persons of enfeebled and cachectic habits, not only have the whole of the structures in which the diseases originated been rapidly involved, but the heart and thoracic aorta have also become affected. Hodgson relates cases in which pulmonic inflammation was accompanied by aortitis of the most acute and fatal description: and the writings of Bertin and Bouillaud are replete with similar instances. The aorta appears, also, to have the same tendency to participate in acute disease in some of those cases of phthisis which are so strongly marked, at once, by entire prostration of all healthy reparative power, and by extreme liability in the system to take on inflammation of an asthenic kind.

In the early stages of aortitis, few of the structures implicated become the seat of inflammatory deposit. When diseased action has existed longer, the cellular tunic may become œdematous. The middle tunic is liable to softening, which diminishes its tone and exposes it to rupture. But the membrane lining the aorta, and the structures immediately subsidiary to it, are liable, when acutely inflamed, to present one of two alterations; each forming a disease strongly characteristic of the constitutional state which engenders it.

*First Type.*—This appears to result from the accession of a very active form of adhesive inflammation; in which a great tendency to the deposition of plastic matter is shewn, by the presence of a single layer of coagulated lymph, of variable thickness, remaining attached over a greater or less surface of the serous membrane. We are, however, but rarely able to discover this kind of effusion in its original form of a single fibrinous layer, (excepting when the disease proves very rapidly fatal, or the patient expires suddenly from other causes,) on account of the great tendency which the blood has to deposit masses of coagulum upon any inflamed surface with which it comes in contact. A condition closely similar to this, in the smaller vessels, appears to give rise to most of the cases of dry gangrene, as it affects the extremities of aged persons, We have sufficient evidence for stating, that in this disease, before the arteries of the affected limb become plugged with coagula, their linings, already rendered scabrous by old disease, become clothed with a plastic effusion, the product of acute inflammation: this deposit forms a medium by which the organizable portion of the clots is retained in the tubes until the separation of the sphacelated part is effected. The manner, however, in which the product of inflammation is disposed upon the diseased membrane appears not to be precisely the same in the smaller branches as in the primary arterial trunk; for while one side only of the calibre of the aorta usually presents a coating of lymph, in the less tubes, the adhesive matter is poured out more equally over the whole interior of their canals. We occasionally observe large masses of pale fibrinous matter very closely adherent to some portion of the aortic walls; especially, either about the sygmoid valves, or in the abdominal portion of the artery, an inch or two above its furcation, partially obstructing the canal, and sometimes (when in the latter position) entirely closing it. From the marked traces of inflammatory change in other parts of the vessel, which usually accompany this lesion, some authorities surmise that these masses are entirely produced by the diseased internal membrane: but it must be submitted, that although their bases or nuclei may have had an inflammatory origin, their larger portion is probably composed either of pale fibrin, similar to that found after death in the cavities of the heart, or of mere coagula of blood, which, having slowly collected over a surface rendered irregular by the presence of a thin false membrane, or by a villous state of the lining itself, have gradually proceeded to assume a large size, and often a semi-organized appearance. Although these productions must necessarily tend to impede the flow of blood through the vessel, and do, in fact, usually produce a tendency to gangrene of the lower extremities, it is very remarkable for how long a time vessels thus obstructed will sometimes continue to take part in the circulation. It occasionally appears, that the inflammation which precedes the formation of these plugs ceases spontaneously; and the masses become permanent, contract, and receive earthy or other deposits.

After noticing some specimens, Dr. Chevers continues,—That form of aortitis, in which layers of coagulable lymph are deposited upon portions of the internal membrane of the vessel, appears to occur under one of two states of the circulation; or it may result from their combination. It may arise, in the first place, while the flow of blood through the vessel is feeble, or unnaturally tardy, from diminution of the heart's impelling power. Thus it is observable, that in some cases of exhaustion during the latter stages of adynamic disease, inflammation of a low type is set up in the aorta; and lymph becoming effused from portions of its lining, the current of blood is not sufficiently strong, either to prevent the formation of a false membrane, or to wash it from its attachment when produced: and, secondly, it may occur where, from some actual impediment to the complete emptying of the vessel, a degree of stasis of the current takes place in the aorta; and the products of any inflammation, then induced, are allowed to collect upon its membrane at any diseased point above the obstacle. During certain stages of Bright's Disease, the lining of the aorta appears to have a great tendency, in common with all the serous membranes, to suffer from inflammatory effusion; and is then liable to receive deposits of coagulated blood, or of adhesive matter upon its surface. The formation of these is probably due to the impediments which the blood receives in the aorta; from the obstructed condition, almost invariably existing in these cases, of the branches terminating the renal, splenic, and hepatic arteries; together with the languor of the heart's action produced by the œdematous state of the lungs, and the tendency to coma so frequently present during the latter hours of patients sinking under this disease.

*Second Type.*—The second class of inflammatory changes met with in acute aortic disease bears a close resemblance to the worst form of erysipelatous action; not only in the rapidity of its progress and the character of its products, but also in its selection of the most debilitated and cachectic individuals, as the subjects of its ravages. It occurs only in persons of the most debilitated constitutions, or in those worn out by the pressure of long-continued disease. It appears either during the latter stages of ataxic fevers; or in the subjects of chronic visceral disease, suffering under the effects of severe operations, profuse hæmorrhage, phlebitis, or arteritis. As the matter effused from the affected surfaces is not sufficiently plastic to resist the current of blood, neither lymph nor coagula form upon the interior of the vessel; and thus the morbid action continues to spread onward, until nearly the whole of the great arteries are involved in the fatal mischief. This state appears to be identical with a somewhat rare form of disease in the smaller arteries—the erysipelatous arteritis; which Cline and Abernethy saw induced by the mere application of ligatures, in the operation for aneurism; and which also occasionally results, either from very slight injuries to the extremities, or, idiopathically, from causes purely constitutional. Here the truly reparative process, by which lymph is effused upon the walls of an inflamed vessel, giving rise to the coagulation of blood within it, is not effected: no bar is formed to the progress of the disease; gangrene of the limb does not occur; but the inflammation ceases not to advance, until it has reached the aorta and the heart.

Dr. Chevers takes up the question, how far discolouration of the arterial linings is occasioned by imbibition. To determine this he has made several experiments. From them he infers:—

That, during cold and temperate weather, stains from imbibition will not be produced in tolerably healthy arteries until after the usual time at which post-mortem examinations are made.

That stains, very different from those produced during life, result from the imbibition of blood similar to that usually found in the aorta.

Again: That a colour produced by inflammatory action becomes somewhat changed after death, by the long application of fluid blood.

Dr. Chevers goes on to observe that, the most common appearances of reddening in the internal membrane of the aorta, which (where the patients have also suffered from other acute, local, or constitutional disease) are assignable to inflammatory action, appear, in their incipient form, as blushes of a bright pink colour, diffused over parts of the internal membrane, without any evident alteration in its texture. At a more-advanced stage of the disease, we observe the lining membrane assuming throughout a deep crimson hue; which is, in some parts, rendered purple, from the addition of a darker stain after death; it may also be thickened, and have a dull or almost villous appearance: but this is unfrequent. Its tenacity is often diminished, the slightest pressure sufficing to strip it from the vessel; and it is usually raised, in many spots, by solid masses of lymph deposited in the laminated sub-serous tissue; these generally peel off with the internal membrane, and appear, until cut into, like red vesicles rising behind it. I think, that so long as these masses retain their transparency and florid tinge, we may justly infer that acute aortitis has been lately present: for, shortly after their deposition, a slight cloud of white opacity begins to appear in the centre of each, gradually becoming deeper, and extending to the edges: they then acquire an almost cartilaginous toughness, and proceed slowly to undergo the atheromatous or ossific change. Patches of lymph, differing from these only in colour, are not unfrequently found, unattended by any reddening of the internal membranes, in aortæ which have long been subjected to irregular distention, and to its almost necessary result—subacute inflammation. They are occasionally of rather large size, often projecting considerably into the calibre of the tube, and may, at times, be noticed undergoing various stages of change in different parts of the same vessel; those most recently deposited, appearing almost transparent, and of a very light straw or amber colour; while those of older date, either have opacities in their centres, or have assumed, throughout, a dead-white colour, and a nearly cartilaginous density.

Dr. Chevers, in opposition to M. Bizot, believes that these deposits take place into the sub-serous fibrous tissue—and that the cartilaginous patches become osseous.

Passing over some hypothetical suggestions, we arrive at Dr. Chevers's account of the—

*Causes of Aortitis.*—Dr. Chevers thinks, "it may be offered as a broad principle, admitting but few exceptions, that inflammation of the large arteries, and, I may add, of the endocardia, arises either in conjunction with similar states in other membranes of analogous function, or under a condition of the system in which the whole of the serous cavities are rendered peculiarly liable to the aggression of inflammatory disease of the most rapidly spreading kind and the lowest type."

Not unfrequently inflammatory action is propagated from the lining membrane of the heart to the aorta. And it is not uncommon to notice extreme dilatation of the left ventricle, from chronic disease of the sigmoid valves, as well as concentric hypertrophy of the same cavity, attended by inflammatory reddening in portions of the aorta, and by the effusion of recent lymph, in reddish or straw-coloured patches, behind its lining.

Aortitis appears to be a by no means unfrequent sequence of phlebitis. In cases of cachectic patients, where the large veins of the extremities have become suddenly inflamed—either spontaneously or after injury, or from rheumatism, and, far more frequently, when the uterine sinuses have suffered inflammation, shortly after parturition, in females of feeble and irritable diatheses—all the large communicating venous tubes, and the cava itself, have participated in the mischief, conveying it rapidly to the heart: and here not only have the right cavities of the organ presented the worst forms of inflammatory change, but the pulmonary artery and veins have suffered to a similar degree: and Bouillaud relates a singular case, in which phlebitis extending to the right side of the heart and pulmonary artery, the aorta presented traces of recent inflammation, while the lining of the left cavities was pale, and offered no evidence of disease.

Acute rheumatism has not been known to give rise to fatal aortitis, but a dangerous form occasionally ensues where a transient attack of acute rheumatism has given rise to that intense form of synovitis which has been termed "purulent-arthritis" by Dr. Stokes—a disease marked by effusion of pus into one, or perhaps several, of the large joints, together with the removal, by ulceration, of the synovial membrane, and, occasionally, with the rapid destruction of the articular cartilages: the arterial systems of patients so affected, have been found implicated in the worst degree. Severe aortitis, too, not unfrequently co-exists with secondary inflammation of the serous membranes.

*Symptoms of Aortitis.*—Dr. Chevers' symptomatology is not very satisfactory. He says:—

"The general train of symptoms which mark aortitis (as derived from many cases I have collected, together with several I have myself seen) appears to be this:—A patient of cachectic habit, and perhaps subject to some form of passive hæmorrhage, becomes suddenly affected with acute thoracic inflammation, attended by more or less prostration of the system, with a scarcely-accelerated but sharp and small pulse, and somewhat hurried and difficult respiration. These symptoms, having been preceded by rigors, usher in an attack of acute inflammatory fever, with general uneasiness, presently amounting to a state of extreme irritability; pain, occasionally of great severity and tearing character, in the præcordial and abdominal regions, along the course of the spine (a sign of importance, inasmuch as those forms of pleurisy and pericarditis which usually complicate aortitis are rarely indicated by continued pain): there is now a tendency to syncope, alternating with restlessness, great heat of skin, a furred and vividly red condition of the lips and edges of the tongue, unquenchable thirst, and a rather full and rapid pulse, with tumultuous action of the heart, imparting a sensation of *fremissement* to the hand applied over the præcordia. After a time, these symptoms are succeeded by the signs of collapse: there is now extreme prostration; the features either shrink and become sharpened, or are bloated and livid; the surface is cold and discoloured; the pulse rapid and indistinct; the breathing difficult, to orthopnœa, or it may become stertorous; the patient falling into a state of coma, from the occurrence of effusion into the base and cavities of the brain: the extremities swell, their superficial veins shewing, through the skin, in dusky lines of ecchymosed appearance: the respiration is at length performed only by sudden gasps at long intervals; and the patient dies with the general aspect of a person suffering from the absorption of an animal poison.

Such are the symptoms which mark the typical form of the disease; but, in the majority of cases, very few of them are present. Thus, a patient sinking from the effects of erysipelas, of extensive suppuration, of phthisis, or, again, from the irritation induced by a surgical operation, may be the subject of extensive aortic inflammation, and still have few symptoms superadded to his condition of prostration and general constitutional disturbance beyond some dyspnœa, together with an increased degree of irritability and distress: so entirely ataxic are some of the forms of acute disease, to which the aorta is liable. Dr. Bright, however, mentions an important sign, which he noticed in three cases of aortitis; namely, the existence of a state of morbid sensibility so intense over all parts of the body, that merely pressing the wrist of one of the patients caused him to cry out with pain."

But the disease may escape observation in the last stages of a typhoid fever, or in common with mere thoracic inflammation, or in those who have sunk under low forms of the exanthemata. So that our readers may well suppose that aortitis is more often recognized on the dead than on the living body.

*Treatment*.—The admission, on the part of Dr. Chevers, that the disease is a desperate one, is also an admission of the usual futility of treatment. Indeed we may safely venture to say that the disease is as difficult of treatment as of diagnosis. Dr. C. thinks that the occurrence of aortitis, and its concomitant inflammations, might frequently be obviated, by constant attention to the state of the secretions of those who appear to be liable to these attacks, at the same time allowing a proper and nourishing diet, with the temperate use of stimulants where they have long been taken habitually. And it is probable, that surgical patients, in great metropolitan hospitals, would generally escape these attacks, if, before being submitted to operations for the removal of chronic local disease, they were kept for a few weeks upon a moderately-strengthening diet; and were it to become an established rule with surgeons, to defer, if possible, the performance of any operation upon individuals, suffering either from hepatic disorder, or from those states of renal disease which are indicated by albuminous urine, until these conditions were removed, or mitigated, by the usual medical treatment. Dr. C. conceives that, in the commencement of aortitis, the milder preparations of mercury may be given in *small* proportions, combined with large doses of opium or hyoscyamus, providing the patient remains perfectly free from symptoms of cerebral mischief. Again: Where there is evidence of great excitement of the circulation, remedies tending more immediately to tranquillise the heart's action, as digitalis or the acetate of lead, may be employed in combination with opiates.

These means will be aided by the application of counter-irritation to the chest and back; but not to a degree sufficient, either to produce severe lesion of the integuments, or to add to the general irritation in the system. The infriction of tartarised antimonial ointments, as recommended by Dr. Copland, together with dry-cupping, are not contra-indicated; whereas the use of blisters, issues, and setons, is highly objectionable, on account of the feeble power which the constitution possesses, during the continuance of the disease, to repair structural lesions, even of the slightest kind.

In the latter stages of aortitis, the frequent administration of small quantities of the stimulants to which the patient has formerly been habituated, or of ammonia and other agents calculated to fulfil the same intention, with the application of warm and stimulating liniments to the chest, will nearly comprise the therapeutics of this disease.

Such are the views of Dr. Chevers. With the utmost deference to observation so careful, and precision so great, we still feel incredulous of the amount of aortitis reputed to exist by Dr. Chevers, and not quite satisfied with the nature or amount of the evidence adduced. Probably we are wrong, but we cannot help suspecting that much of what goes for aortitis is the consequence of imbibition in the later hours of life, or after death.

### V. Cases of Malignant Disease of the Lung. By H. Marshall Hughes, M.D.

Dr. Hughes remarks that, notwithstanding the greatly increased attention which has been latterly paid to the investigation of thoracic complaints, and the exactness with which, by the aid of percussion and auscultation, the existence of many affections may be predicted, the diagnosis of malignant disease of the lung remains in a great measure uncertain. The absence of any definite symptoms, and the indeterminate character of the physical signs—or the limited opportunities of ascertaining them, in consequence of the comparative rarity of the complaint—have hitherto rendered its detection a matter of difficulty and of doubt. It may indeed still be said, in the words of a recent writer, "As yet, almost nothing has been done, in establishing the diagnosis of this disease."

Dr. Stokes has mentioned two forms of the complaint. In one, "degeneration of the lung occurs, and the organ is transformed into a cancerous mass. In the other, a tumor is formed external to the lung, which it ultimately displaces as it progressively increases in size. He has not noticed, or only slightly alluded to, a third, and, according to my own observation, a very much more common form than either of those to which he has referred. In these cases, rounded masses, varying in size from small marbles to small oranges, white, pink, or purplish, in colour—and solid and semitransparent, or friable and opaque, according to the age or character of the affection—are found distributed throughout the greater part of one, or, more commonly, of both lungs; while disease of a similar kind, but in a more advanced stage, exists in some other organ or organs of the body, as the mamma, uterus, testicle, kidney, or liver, or in the bones and soft parts of the extremities. When, indeed, fungoid disease has long existed in any part, and at the period of the patient's death has already made considerable progress, it is far from uncommon to find, on dissection, masses of the character alluded to in the lungs, as well as in other internal organs.

Dr. Hughes introduces four cases. The first of these is a—

*Case of Fungoid Disease of the upper part of the Lung.*—Mary Benbow, aged 50, a washerwoman, admitted August 19, 1841.

Two years ago, she caught cold, and was confined to her bed for two months; ever since which she had been subject to occasional attacks of hæmoptysis. She had been under Mr. Kingsford's care for nine months; and during that time had been several times attacked with spitting of blood, for which, on two occasions, she was bled; but was generally treated by the administration of acids, acetate of lead, digitalis, and saline purgatives. When admitted into the hospital, the countenance was rather pale and sallow, with a few enlarged cuticular veins in the cheeks; her legs were swollen; she had no pain, nor was she particularly emaciated; she lay upon her back, with the shoulders rather raised, and somewhat inclined to the right, but could turn to either side, or get up without much inconvenience; she complained of cough, accompanied with shortness of breath and sanguineous expectoration; her tongue was slightly coated, and moist; her skin soft and unctuous; her pulse frequent and feeble; her bowels regular. The sputa, on examination, were found to consist of white frothy mucus, with some portions of a light crimson colour from their perfect admixture with bright-red blood. She had one absorbent gland, nearly as large as a pigeon's egg, in the right axilla, and a smaller one under the right clavicle; but had not been aware of their existence till they were pointed out to her. The superficial cutaneous veins of the right side of the abdomen, and the lower part of the chest, were considerably increased in size, and rather tortuous.

*Physical Signs.*—On inspection of the chest, it was at once evident that there existed a very decided sinking and flattening below the right clavicle, extending nearly to the mamma; that the ribs of the part were very little moved in respiration, and that when elevated, however slightly, they were raised *en masse* as a solid unyielding case. Over this portion of the chest, at the upper part of the right axillary region and over the right scapula, there existed a perfect deadness of sound on percussion; complete loss of the respiratory murmur; very marked tubular or tracheal respiration, only rarely accompanied by a little coarse mucous rattle; great shrillness and loudness of the voice, approaching to imperfect pectoriloquism: and obviously increased tactile vibration. The morbid phenomena appeared to terminate at a defined line just above the mamma, and to pass round the whole of the right side of the chest. The lower portion of the right and the entire left lung appeared healthy.

It is unnecessary to enter any notice of the treatment adopted, as only palliatives were employed. Her symptoms varied but little: she had occasionally severe pains of the left side, which were relieved by sinapisms; her dyspnœa became gradually worse, and soon amounted to orthopnœa: the œdema of the legs increased. She had no ascites, and, while in the hospital, had no hæmoptysis of any severity. The physical signs remained of the same character, but extended downwards with considerable rapidity. Without any particular suffering, or very great emaciation, she died exhausted, Oct. 17th.

*Dissection.*—The left pleura was slightly adherent, from old standing disease. The left lung was crepitant throughout, and partially emphysematous. The right pleura was universally firmly adherent, and superiorly altered in texture by a white flaky malignant deposit. The entire upper part of the right lung was converted into a mass of medullary fungus, the cut surface of which exhibited a dead-white cheesy substance, intersected with bands of cellular tissue. By slight pressure, a creamy fluid exuded, together with portions of soft brainlike matter, from cells varying in size from a pin's head to a marble. The middle lobe contained some portions of the malignant growth, appearing like elongations or processes of the diseased mass above them, from being clearly connected with and traceable into it and separated from each other by the intervention of healthy or simply-compressed lung. The interior lobe contained a few small detached masses of fungoid matter, and was, posteriorly, firm, dark-coloured, and lacerable, probably from gravitation. In the branch of the right pulmonary artery, going to the upper lobe, there was a small pedunculated medullary tubercle, and another on its external surface. The heart and pericardium were natural.

We pass over the *second case*, although not uninstructive, but pause at the third.

*Case of Fungoid Disease of the Thigh and Lungs.*—Sarah Swaisland, aged 14, admitted into Guy's Hospital, under the care of Mr. Callaway, Jan. 6th, 1841.

About a year ago, she received a kick from a boy upon the knee; and a short time before admission, the joint became swollen and painful. When she entered the hospital, it was inflamed and tender, but not much enlarged. It increased, however, very rapidly in size, and amputation was proposed; but not assented to, till it was impracticable. Her pain was relieved by morphia: but neither topical applications nor internal medicines had any effect in retarding the growth of the tumor. She died June 1st, 1841. Her chest was not examined during life, as she had no constitutional irritation, no cough, difficulty of breathing, or hæmoptysis, to induce her attendants to suppose that she had any disease of the lungs.

*Dissection.*—The body was emaciated, and the mammæ but little developed. The great tumor consisted of radiated, long, and fungoid tissue, very, but variously, fleshy and vascular; soft, and but little or nowhere cerebriform; and in little parts, escaped from within the periosteum. The joint was but little inflamed. There was suppuration about the groin: both legs were more or less œdematous: a turbid effusion in the periosteum. The liver was large, coarse, and soft; or so flabby as to give the idea of a sac of fluid. It was not very lacerable, but darkish. The lungs contained numerous tubercles, about the size of peas and chesnuts, firm, roundish, nodular, semicartilaginous, somewhat translucent; and some were very earthy. The heart was small.

A case like this, shews, as Dr. Hughes observes, that numerous masses of fungoid or schirrhous matter may be deposited in the lungs, while a similar disease is advancing with greater rapidity in some other part or parts of the body, without causing any symptoms of pulmonary irritation. The attention of the observer is, consequently, not particularly directed to the state of the chest; it is not at all, or only very superficially, examined; and the disease of the lung is often not even suspected, till displayed upon the inspection-table.

All who have seen much of cadaveric examinations have seen instances of this sort. We frequently hear of patients dying *consumptive* who have been operated on for cancer. No doubt, malignant disease of the lung is mistaken frequently for phthisis, and practitioners would do well to peruse these cases and prepare themselves for similar occurrences.

After relating another case of dubious character, Dr. Hughes sums up:—

"I presume the two first cases herein related are genuine and characteristic specimens of what Dr. Stokes calls 'cancerous degeneration' of the lung. If, then, the two last cases are excluded from consideration, and a review taken of the two first in connexion with the two of a similar form of the disease recorded by that experienced physician, it will be found, that, in all four, the disease existed on the right side—that all were more or less troubled with hæmoptysis—that, in three out of the four cases, the sputa, which in the fourth are merely stated to have been tinged with blood, presented a very peculiar appearance, Dr. Stokes having compared them very curiously to 'black-currant jelly,' while I have frequently publicly noticed their resemblance to 'red-currant jelly mixed with water'—that in all there existed evidence of obstruction in the superficial veins of the affected side, evinced either by the enlargement of the vessels themselves, as in three, or by œdema of the parts situated, in reference to the progress of the venous current, just below the diseased organ, as in the fourth—and that, in two, tumors were discovered in other parts of the body. It will also be found, in reference to the physical signs, that there was, in all, perfect dulness on percussion; absence of the natural vesicular murmur; and tubercular or tracheal respiration, without any rattles, or with only a little, of a simply bronchial character. We may, perhaps, therefore conclude, though there are no physical signs at present known which are peculiar to or are pathognomonic of this complaint, that when there exist signs of extensive solidification of the lung, without either the previous history of pneumonia, or any evidence of softening of the morbid product—when the patient has been troubled with hæmoptysis, and the general symptoms and progress of his affection are inconsistent with the presence of tubercles—when the sputa occasionally consist of blood, so thoroughly incorporated with serous fluid as to resemble currant-jelly—and when the veins of the neck, arm, chest, or abdomen, on the affected side, are enlarged, or there is local œdema proving obstruction to the flow of blood within them—a suspicion may be entertained of the existence of malignant disease of the lung; and that this suspicion will be strengthened by the complaint being situated on the right side, and especially by the appearance of tumors in other parts of the body.

It must however be added, that the disease sometimes, as in the second case herein related, very accurately resembles empyema—that the history of the case, and the physical signs, are on such occasions insufficient for the purpose of distinguishing the two complaints—and that the diagnosis, if at all practicable, must then be deduced from the general symptoms, the peculiar character of the expectoration, the obstruction to the flow of blood through the superficial veins of the affected side, and the appearance of malignant tumors in other parts."

It is our impression, although we should be sorry to speak very positively to the point, that we have seen at least one instance of medullary tubercles in the lungs unattended with the expectoration described by Dr. Hughes. Unfortunately, we have lost our notes of the cases, and can only speak thus loosely from memory. The subject merits close observation. It is pretty clear that when a patient, who has suffered from malignant disease in any organ, complains of thoracic symptoms, suspicion should be pointed to the probable occurrence of malignant affection of the lungs. Dr. Hughes has done well to draw more general attention to the subject.

### VI. Reports of Cases requiring Capital Operations which have been performed, since October 1, 1840. By Bransby B. Cooper, Esq. F.R.S.

The following list is subjoined.

### OPERATIONS IN CASES OF IMPORTANCE, PERFORMED IN GUY'S HOSPITAL, BY MR. B. B. COOPER, FROM OCT. 1840 TO JULY 1841.

For *Aneurisms* 4 in Guy's Hospital, : 1 in private practice:

| | | |
|---|---|---|
| Of the | Subclavian . . . | 1, unsuccessful. |
| | Popliteal . . . . | 3, all successful. |
| | Common Carotid . | 1, successful, (in private practice.) |

*Lithotomy*—5; viz.

| | |
|---|---|
| In Children . . . . . | 3, all successful. |
| In Adults . . . . . . . | 2; one successful; one unsuccessful. |

*Excision of Elbow-Joints*—2, both successful.

*Excision of Cartilage from Knee-Joint*—1, still doubtful.

*Amputations*—8; viz.

| | | | | |
|---|---|---|---|---|
| Of Upper Extremities . . | 5 | for disease, | 2, | sucessful, 1. |
| | | | | unsucessful, 1. |
| | | for accident, | 3, | all successful. |

Of Lower Extremities . . 3 { for disease, 1, successful. / for accident, 2, both successful.

*Removal of Diseased Breasts*—3, all successful.

*Trephining for Injury*—1, unsuccessful.

*Removal of Tumors*—4; viz.

| | |
|---|---|
| Steatoma from over Scapula . . . . | successful. |
| - - - - - - - Deltoid . . . . . . | ditto. |
| Suspicious tumor from Elbow . . . . | ditto. |
| Steatoma from the Gluteal Region . . | ditto. |

*Fungoid Testicle,* removal of—1, successful.

The cases are reported at length, perhaps a little too much so, and we must refer our readers to the originals. We shall content ourselves with noticing a point or two.

*Mode of passing the Ligature for Popliteal Aneurysm.*

In a case in which Mr. Cooper tied the femoral artery, instead of the usual mode, when the artery is exposed, of drawing the saphenous nerve outwards, and then passing the aneurismal needle, armed with the ligature, between the artery and vein, Mr. Cooper adopted the following plan:—immediately on opening the sheath, he passed an aneurismal needle, unarmed, below the whole of its contents, from without to within, and to such a distance, that its curved extremity appeared close to, and on a level with, the upper and inner edge of the artery. There were two branches of nerves in this case, running along the upper surface of the artery: having gently detached these, by means of the blunt edge of a small probe, Mr. Cooper "tilted" them over the exposed end of the aneurismal needle; on which, therefore, remained lying only the artery and vein. Between these, Mr. Cooper now insinuated the end of the probe; and through the space thus made, passed the needle, having removed it cautiously from its former situation. In this situation, the needle was armed by an assistant, and the ligature then secured in the usual way. This method appeared to place the separation of the artery from the nerves and the vein more under the command of the operator, while it caused the least possible disturbance to its cellular attachments.

"I may remark," says Mr. Cooper, "that the position in which the thigh is placed before the operation is of great importance, in reference to the subsequent passage of the ligature. By the full rotation of the thigh outwards, and by the bending of the leg, all the muscles are relaxed; and by a pillow being placed under the leg, the patient is freed from the apprehension of moving the limb, and thus interfering with the operation. In regard to the direction of the incision necessary to expose the artery, there is no doubt that, as a general rule, the inner edge of the sartorius muscle is the best guide; but frequently in cases, where, from œdema extending over the thigh, this edge cannot be distinguished, a line may be taken from the centre of Poupart's ligament to the inner side of the patella, and the point at which it is intersected by another line, taken from the anterior superior spinous process of the ilium to the tubercle of the inner condyle of the femur, marks the spot at which the ligature should be applied. The artery, in the case in question, was, as before narrated, seen pulsating on its exposure; but I may state—though to many the remark will be superfluous—that this is far from being uniformly the case. I have seen even the carotid lie so perfectly quiescent after exposure, that a surgeon has thought, from the total absence of pulsation, that it could not be an artery. The whitish-coloured vessel which is exposed in these operations is only discovered to pulsate by being pressed between the finger and thumb. From the details in Case No. 2, I think that the advantage of passing the needle before it is armed with the ligature, is sufficiently shewn. It necessary to take great care that the point of the needle be not too sharp; or there will be considerable danger of wounding the vein, whilst the needle is being passed between it and the artery. As so the tightness with which the ligature should be drawn, the surgeon should exercise his discretion, and is best taught by experience. It is frequently stated, that the inner and middle coats of the artery should be felt by the operator to give way under the ligature; but, in a very great majority of cases, I have not been able to detect any thing of the sort. In old persons, where the coats are more likely to be indurated or ossified, a less degree of force will of course be proper. In Cases Nos. 2 and 3, the patients were sensible of an injury at the moment at which the aneurism may be supposed to have originated; and the same remark applies to the following case, No. 4. This is an unusual circumstance."

### Case of Ligature of the Common Carotid Artery.

The Rev. Mr. ———, aged 34, a native of Barbadoes, having lately arrived from that island, made application to Mr. Cooper, on June 30th, for the cure of an aneurismal tumor on the right side of his face.

The tumor was about the size of a small walnut, deeply imbedded within the substance of the parotid gland, and placed close to the neck of the lower jaw, apparently in the exact situation of the division of the external carotid into the temporal and internal maxillary arteries. Its pulsation was very perceptible to the finger; but not so readily to the sight, from its being covered by the parotid gland. On applying the ear, the peculiar whiz characteristic of aneurism was distinctly perceptible. By pressure on the common carotid artery, not only this, as well as the pulsation, ceased, but the tumor itself entirely disappeared: the pressure being removed, it most rapidly resumed its former position and appearance. On depression of the lower jaw, the prominence of the tumor was no longer discernible. This state of things had led to no inconvenience whatever to the patient; for he apparently performed every function as naturally as if entirely free from disease.

The tumor had first been noticed accidentally about a twelvemonth previously. Mr. Cooper, as well as Sir B. Brodie and Mr. Liston recommended an immediate operation. It was performed at half-past two P.M. on the 7th of July.

An incision, about two inches in length, was made, commencing on a level with the middle of the thyroid cartilage, along the inner side of the sterno-cleido-mastoideus muscle, but nearer to the median line than usual. A small superficial artery was divided and secured: there was not much venous hæmorrhage. A quantity of cellular tissue being divided, the omo-hyoideus and sterno-hyoideus muscles were exposed; and the artery was felt pulsating at the bottom of the wound, which appeared very deep. The omo-hyoideus being then drawn downwards and inwards by means of an aneurism-needle, and the neck a little relaxed so as to bring the parts better into view, the sheath was cautiously opened, as much to the inner side as possible. The artery was thus exposed. There was no obstruction from any swelling up, or over-lapping of the jugular vein: which did not, in fact, present itself to view. An aneurism-needle, unarmed, was then passed beneath the artery; upon which, at the time, a small nerve was lying: and when this had been carefully detached, for a small distance, by means of a blunt silver probe, the needle was armed with the ligature. One end of this was now passed between the upper surface of the artery and the small nerve already spoken of; and this being held securely, the other end was drawn out, together with the aneurism-needle, from beneath the under surface of the vessel. The effect produced on the tumor by pressure on the artery was observed; and being found to be satisfactory, the knot was made. The aneurismal sac immediately became flaccid, and yielded entirely to pressure. The wound was closed by two ligatures and adhesive plaster; and the patient placed in bed, with his head raised and neck relaxed.

During the operation, he displayed unusual fortitude and submission; neither disturbing the parts about the throat by uttering exclamations, nor offering resistance by struggling. Towards the latter part of the operation, prior to the ligature being put round the artery, he once or twice coughed, with a peculiar barking sound; but this was evidently involuntary.

The right side of the face was cold for that day. Every thing went on well. The ligature did not separate until the 10th of August, when the report goes, that the facial and temporal arteries on the right side of the face appear to be obliterated. There is no perceptible difference in the vessels on the opposite side.

The patient mentioned a curious circumstance. He asked if he had been observed to make during the operation, a curious noise, like the bleating of a goat; and said, that it was quite involuntary, and was attended with a curious sensation, as of some one squeezing the lower part of his throat, and also a sudden sense of weight at the stomach and a disturbance in the bowels; so that he was, for a minute or two, afraid he should not be able to retain his fæces. These sensations, together with a general sense of faintness, occurred at the same instant; and, he said, seemed to depend on Mr. Cooper's pushing aside something with his finger in the wound. No doubt this was the nervus vagus. Mr. Cooper makes a remark on the method of operating, which we think is of importance.

"The patient being placed in the recumbent position, I commenced my incision more to the inner side, or towards the sterno-thyroid muscle, than is usually recommended; with a view, upon opening the sheath, of more immediately exposing the carotid artery; being convinced, from examination, that the usual direction given to cut down along the edge of the sterno-cleido-mastoideus muscle leads rather to the jugular vein than to the common carotid artery. This conviction seems verified by the fact, that most authors, in their description of this operation, speak of its difficulties principally arising from the distention and rising of the jugular vein, a circumstance which not only did not occur in this instance, but the jugular vein was not even brought into view, nor did it in any way interfere with the application of the ligature. The only difficulty I met with, was from the depth at which the vessel was placed from the surface, and of which the appearance on the dead subject offers no criterion. Having made the opening into the sac so much on the inner side for reasons already given, this depth of the vessel offered impediment to the ligature being passed, as usual, from without inwards, but readily permitted its passage from within outwards; a mode which is equally safe, if the forefinger of the operator is placed so as to protect the vein from any injury from the point of the needle. I armed the aneurismal-needle, as I always am in the habit of doing; as the needle passes so much more freely without than with the ligature. The patient seemed not to express pain from the tightening of the ligature at the moment; nor did he afterwards exhibit any indications of suffering from the sudden change of circulation to the brain."

Two cases of Excision of the Elbow Joint are reported. Mr. Cooper observes upon the subject:—

"The operation is indicated when it is evident that nature can only effect a cure by anchylosis, and when at the same time it is plain that the constitution is unequal to endure the prolonged and exhausting irritation attendant upon the exfoliation of bone and protracted discharges. Of course, the more the disease has been confined to the structures of the articulation itself, and the less the bone has suffered, the greater will be the probability of a successful result.—I may here remark, that in operating upon the elbow-joint, the inner incision should be carried close to the olecranon process, so as to run no risk of injuring the ulnar nerve, which should be then drawn inwards by a metal retractor. It will not be injured by this step; being protected by the adipose tissue, in which it lies imbedded, and by which, indeed, it is completely hidden. The outer incision should be made sufficiently distant from the olecranon to leave the extent of space required for the removal of the diseased parts; which is much facilitated by flexing the elbow-joint after the transverse incision has been made. It has been recommended to divide the triceps muscles from the olecranon preliminarily, in order that, after the latter has been removed, it may be re-united with the ulna; but I do not think that such a union can occur, except by means of adventitious matter, which in all cases is deposited, and which is always sufficiently firm and continuous to maintain the function of the muscle, and protect the posterior part of the new joint.

With respect to the results and general bearings of the operation, I think the principal benefit derived from it is, that it lays open and perfectly exposes the affected tissues, and thus facilitates their separation; in order to effect which, Nature would otherwise have to form sinuses and ulcerations. The advantage gained by it is better estimated by the extent of surface exposed, than by the quantity of bone removed; no more of which should be brought away than is evidently affected by disease, which we shall generally find, is confined to the articulating surfaces."

CONCUSSION OF THE BRAIN is thus alluded to by Mr. Cooper. "When one or both pupils remain contracted, I am induced, from my experience, to consider this as an unfavourable symptom, characterizing lesion of the brain. In those cases in which I have observed contraction of one pupil, and have had an opportunity of making a post-mortem examination, I have invariably found injury of the brain on the side opposite to that of the contracted pupil. The same violence which produces concussion may cause fracture of the base of the scull: and although the constitutional treatment employed may subdue the symptoms of concussion, during the progress of reparation, effusion may take place, and evidences of compression supervene. Such a complicated injury, however, is generally denoted by bleeding from the ear, at the time of the accident; and I have known a discharge of serum from the external meatus continue for many days after the accident; and yet these cases ultimately did well. Even when this discharge is profuse, it is to be regarded as a favourable symptom, and therefore should not be checked by astringents. The case above alluded to was treated successfully, by general and local bleeding and calomel."

*Case of Steatomatous Tumor removed from the Gluteal Region.*

John Baldwin, aged 24, a healthy-looking man, by occupation a labourer, was admitted on May 25th, 1841, under Mr. Cooper, for a large tumor in the gluteal region.

*History of Case.*—He states, that about nine or ten years ago he first discovered a tumor, which he says was then of the size of a pint basin: previous to this it had not attracted his attention. However, as it gave him no pain, he made no complaint, till between two and three years ago, when he consulted a country surgeon, and was taken under his care. After having been in a provincial hospital about three weeks, an attempt was made to remove it, by two elliptical incisions: but the surgeon, during the operation, imagining that it had connexion with the pelvic viscera, desisted from the operation, and closed the wound. In about ten weeks afterwards he went home, and there pursued his usual occupation for two years; but perceiving that since the operation the tumor had greatly increased in size, he became anxious on the subject, and presented himself for admission into Guy's Hospital.

*Present Appearances.*—The tumor now is very large, and perfectly uniform: an impulse is communicated to it by coughing; but the patient states that no change is produced in it by passing the fæces, or by any alteration in his position; and it does not increase from exercise. No fluctuation can be discovered. Great difference of opinion was entertained, as to the diagnosis of this case: by some it was considered as an encysted tumor; by others, as a hernia, escaped through the ischiatic notch. Mr. Cooper wrote to the surgeons concerned in the former operation. The information obtained was not considered as conclusive against a second operation. Mr. Cooper, then, with the concurrence of Messrs. Key and Morgan (the man being extremely anxious to have the tumor removed), determined upon an exploratory operation. On Tuesday, July 28th, the man was placed in the prone position upon the operating table; and an incision was made into the tumor, about three inches in length. The several layers, covering the tumor, which lay beneath the glutei muscles, were successively divided; and the nature of the tumor then became apparent. It was, in fact, a large steatoma, and had been covered entirely by the gluteus muscle, which was expanded over it. It was dissected entirely out, and was found connected with the pelvic fascia.

The man endured the operation, which lasted about half an hour, with the greatest fortitude. There was very little hæmorrhage at the time. The wound was closed by two sutures, and the man was put to bed. About four or five hours after, there was some little bleeding, proceeding from several small vessels: of these, four were tied, which appeared effectually to restrain it; a sponge was placed in the wound, and it was closed. In the evening of the same day, the sponge was removed, which was followed by slight hæmorrhage; and two more vessels were secured. The patient had vomited once; but had very slight pain, upon pressure over the abdomen. The wound was closed again by sutures, and warm water-dressing applied. An opiate of the morph. hydro-chlor. was ordered. The case did perfectly well.

### VII. Account of a Patella broken transversely, and re-united by Bone. With Remarks on the Nature and Treatment of similar Injuries. By T. Wilkinson King.

It would seem that this *was* an instance of bony union of a transversely fractured patella. The *points* of Mr. King's remarks are these.

"If the reparative ossification is to be expected mainly on the convexity of the bone, and if this action is regulated by the degree of inflammation, I have to ask, if it be well to subdue the little undue vascularity which quickly follows the injury? And, in order to enforce the reflection, I would almost say, it is desirable to consider the means of exciting and maintaining inflammation on the surface of the patella, rather than strive to obviate the injection, which is too limited, and so superficial as to be peculiarly under the controul of topical remedies.

In the absence of experience, I am not inclined to think that abstaining from the application of cold, or even attempts to excite capillary action, will be often found to succeed in uniting the fragments of a patella by bone: but I do consider that the habitual use of cold, in the main, is a needless, mistaken, and pernicious practice; and that some aid is decidedly to be looked for from an opposite method."

"The limb is doubtless to be kept extended and even, as much as may be inclined to form a right angle with the body: but when our minds turn to the means by which the upper fragment is to be drawn down and confined, it should, I think, be difficult to avoid the conclusion, that every thing in the shape of a close-fitting circular strap must be, comparatively, inconvenient, inefficient, and obstructive; producing needless pressure, requiring excessive violence, and causing mischievous congestion and unhealthful actions.

All these influences may, I conceive, be pretty readily obviated by an application of a modified character—a solid pad, long and narrow, and curved along the upper edge of the patella. A torniquet so formed, capable of well-graduated pressure, and applied somewhat obliquely and firmly, but without any circular or constricting bands, would, I imagine, act more completely, and with the least necessary violence, without congestion, or any needless obstruction to the nutrient and reparative actions.

It is not now needful to inquire what force is requisite to resist the efforts of the extensors to displace the bone. Doubtless it is desirable to employ the least violence that will be efficient and secure; and my conviction is, that very slight force will suffice, provided the muscles are carefully set at rest, by attention to the patient's posture; and kept unexerted, by studious absence from all motion: for the patient's exertions in bed, even with the most precise application of straps, &c. (unless they are so tight as to be very distressing) cannot fail to act upon the injured bone."

### VIII. ON THE STRUCTURE OF THE BLOOD CORPUSCLE. By G. OWEN REES, M.D. Physician to the Northern Dispensary; and by SAMUEL LANE, Lecturer on Anatomy.

The human corpuscle, say the authors of the Paper, is circular in form, flattened, and presents a double concave surface. When viewed obliquely and nearly in profile, a central depression is distinctly observed. Its diameter measures, on the average, $\frac{1}{3250}$ of an inch. The edge will be found to vary much in thickness, as will be afterwards explained. It usually measures about one-fourth the diameter. A front view also shews a concavity, the centre of which is destitute of colouring matter; but no distinct nucleus can be demonstrated in its unprepared state. We have, however, frequently succeeded in bringing it into view, by decomposing the globule by means of water, or of a very weak solution of sugar and water. A drop of blood and a drop of water may be placed close together on a piece of glass, and allowed to coalesce; when the nuclei will be found to collect principally about the lower edge of the specimen: or the method recommended by Hewson may be adopted, which consists in placing some serum, loaded with blood corpuscles, on a piece of glass. The specimen is to be viewed while forming an inclined plane in the field of the microscope; and a drop of water is to be added, in such a way that it may flow from the upper to the lower edge of the piece of glass. In its descent it will be found to alter the form of the blood corpuscle, which becomes more rounded and transparent. The nucleus may now be observed in many of the corpuscles, as they roll down the glass: but we have never seen them, as Hewson describes them, moving like a pea in a bladder, as the corpuscle turns upon its axis. In order to convince himself of the existence of a nucleus, the observer should treat the blood of a bird and of the human subject in the same manner. In the former, he will have no difficulty in seeing and separating the nucleus, and in the latter, by transferring the information thus gained, he will, after some little trouble, be enabled to recognise similar appearances, to his entire satisfaction. As we before stated, many of our most accurate observers—Magendie, Hodgkin, and Lister—have denied the existence of a nucleus in the corpuscles of mammalia; and, as far as we know, no description has been given of it, which would in any way meet our views of its form, size, and appearance. The nucleus of the human-blood corpuscle is composed of a thin circular layer, of a colourless substance. Its surfaces are granular, and its edge uneven. It is only about one half less in diameter than the blood corpuscle itself; for which, no doubt, it has been frequently mistaken. It measures from $\frac{1}{4500}$ to $\frac{1}{5000}$ of an inch in diameter. Its thickness cannot be so satisfactorily stated; it does not appear to be more than one-eighth or one-tenth of its diameter. It may be demonstrated in its moist or dried state, either within its envelope, or separate from it.

Several circumstances have combined to render this nucleus difficult of observation, and which may serve to account for its eluding the notice of so many micrographers. Its thin shape, circular form, and large size, have led to its being mistaken for the blood corpuscle, deprived of colouring matter; while the erroneous notion that the nucleus was a small globular body has not only favoured the misconception, but has led those who have looked for a nucleus of this form to deny its existence altogether. The edge, also, of the nucleus projects so far into the colouring canal, that its defined margin cannot be seen, until this has been destroyed by the removal of the envelope. The envelope in the human subject does not differ in any essential particular from that of the frog. It forms a closed flattened vesicle, the interior of which adheres firmly to the central part of both surfaces of the nucleus, but not elsewhere. The envelope, like the nucleus, is circular, and forms a complete annular canal around it, in which the red colouring matter is situated.

### IX. A Case of Intestino-Vesical Fistula. By Mr. Hingeston.

The patient was a gentleman, residing in the heart of London, of strumous diathesis, and corpulent make. He had pretty good health till the 5th of May, 1835, when he dislocated his shoulder, which was easily reduced. On the 21st of September of the same year, he was seized with acute pleurisy of the right side, giving rise to strong arterial action, inflammatory fever, hæmoptysis, &c. demanding prompt venesection, salines, mercurials and abstinence, for its reduction. And in the same year, on the 2nd of December, in the middle of the night, profuse hæmoptysis occurred; and venesection was again enforced twice in forty-eight hours, with repose in bed, abstinence, salines, and diuretics.

These two attacks, and the loss of blood consequent upon them, sapped his strength, and evidently made him thinner. In the Spring of the following year (1836), he complained of debility; and (May 25th) suffered from passive bronchitis and anorexia, requiring carminatives and warm aperients.

Throughout his life he had always been troubled with diarrhœa, which seemed beneficial rather than otherwise; besides being afflicted with a mucous cough, or occasionally with sub-acute bronchitis. It was during one of these attacks that, in the act of coughing, he ruptured himself on the right side (September 5, 1836.) To this hernia, which was inguinal, a truss was applied, and it was cured.

In January, 1837, he had the influenza in a low form. In April he first complained of painful micturition, which subsided in May. In 1838, the "strangury" returned, with the odour and presence of fæces in the urine. In the course of February and March, the malady became excessive and exhausting. Fæces, in a soluble state, streamed away from the urethra, intermingled with urine and gusts of wind; the natural office of the rectum was suspended, or performed with irregular and untimely calls; and the heart and nervous systems were disordered. The urine, at this time, was acid and albuminous.

The plan of treatment adopted was that which was, with some modification, continued to the last; viz. opiates, repose, a pultaceous diet, washing out the rectum, and a regulated temperature night and day. By these means the misery was alleviated, and the natural passage of the rectum kept clear and pervious, while the false outlet through the bladder was lessened or diverted.

In June of this year, the first collapse took place: it followed upon a violent and uncontrollable irruption of wind and fæces through the urethra, completely upsetting the residue of the strength, and threatening speedy dissolution. The vehemence of the paroxysm was assuaged by successive doses of opium carefully repeated and watched, warm fomentations, diluent diet, and the recumbent posture; followed by ammonia, wine in arrow-root, and sustaining food. The habitual dose of opium, night and morning, was from 30 to 40 minims of Batley's sedative solution.

By diligent perseverance in this plan, the disease was reduced to a state of quiescence. The fæces were passed by their natural course, or but rarely found their way through the bladder, discolouring the urine; and the only evidence of the fistulous orifice continuing unclosed, was the occasional and unexpected explosions of gusts of wind from the urethra. Even these at length ceased, and the year 1838 terminated in a suspension of all threatening symptoms. The constitutional powers, however, seemed to decline.

In February 1840, at a time when the fistulous opening seemed to be closed and the general health was much improved, hæmoptysis again recurred, and a cavity was discovered in the top of the left lung. Depletion was requisite. His strength was prostrated—the irruption of the fæces with the urine recurred—tympanitis was added—and acute peritonitis, unhappily demanding a fresh venesection to twelve ounces, and shewing buffy blood, almost seemed to imply that a perforation from the intestines into the abdominal cavity might be impending. The balance of the circulation was now never restored; for venous congestion was manifested, both by the darker hue of the countenance, and the blueness of the nails. Tympanites never ceased to be present; and though the fistulous opening into the bladder became finally silent in August 1840, yet the evidence of stricture at or about the sigmoid flexure of the colon became every day less and less questionable. Ascites succeeded, and on the 13th of April Mr. Key tapped the patient, and drew off about three pints of fluid. A very feeble re-action developed inflammation along the arch of the colon; under which he sank, April 15th, in the 65th year of his age.

During the anasarca and ascites, the urine was neither alkaline nor albuminous.

*Dissection.*—The body was emaciated, the lower extremities œdematous, and the course, as well as the figure of the colon, could be distinctly traced beneath the integuments, across the umbilicus.

*The Abdomen.*—On dissecting back the integuments, and laying them open, the colon presented the appearance of a large tromboon, amazingly distended, and stretching across the umbilical region, with its sigmoid flexure, equally large, in the left iliac fossa. The space above its line was occupied by an enlarged liver, and that below by the small intestines.

The omentum was shrivelled, and drawn across to the right iliac fossa; where it was adherent to the parietes above the internal abdominal ring (which was not at all dilated on either side, although a hernia was said to have existed formerly). The peritoneum was universally opaque and injected; especially around the point of paracentesis, and also in both iliac fossæ, where the intestines were involved in a mass of adhesions. There was much buttery lymph both in the bason of the pelvis, and smeared over the surface of the intestines: and the intestines were of a very lacerable texture, devoid of scybala, containing only some fæculent matter and much flatus.

The sigmoid flexure of the colon, just above the rectum, the ileum, and the cæcum, with its appendix, were each and all adherent, *en masse*, to the fundus of the bladder, and involved in a general thickening of the surrounding textures.

*Thorax.*—Both the lungs were firmly attached by old adhesions; especially at their apices, which were interspersed with small tubercular excavations about the size of a bean, and lined by (an acquired) mucous membrane. The surrounding parenchyma was crepitating, and but little consolidated. The basis of each lung was crepitant, and healthy.

The bladder, with the adherent intestines, was removed; and a particular dissection made of them, as follows:—

The colon, hypertrophied, singularly muscular, and in circumference about the size of a man's arm, was, together with a convolution of the ileum, and the appendix cæci, adherent to the fundus of the bladder. The natural course of its canal was impeded by a contraction or stricture, which commenced inferiorly in the rectum, about the forefinger's length from the anus (just at the base of the triangle formed by the vesiculæ seminales), and extended upwards for about two inches, barely admitting the entrance of the little finger. A section of the gut in this part resembled scirrhus; and the glands were, with the surrounding tissues, thickened.

Immediately above this stricture, the coats of the bowel were riddled with ulcerations and openings, leading into a channel which separated the bladder from the intestine. This channel was, in fact, a feculent abscess, situated beneath the reflected portion of the peritoneum, between the bladder and bowel. It was degenerate in structure, lined with a dark membrane, and filled with a muco-purulent excretion, It opened, anteriorly, into the fundus of the bladder; above, into the colon; below, into the rectum; and posteriorly, through the colon into the ileum: so that there was a false passage, by which the natural course of the colon was diverted, and forced between the bladder and strictured part of the intestine down into the rectum below, and at the same place, by means of a fistulous opening, into the bladder in front. The orifice of this fistulous opening within the bladder was curtained by a fungous growth or thickening, which overhung it like a valve. Thus, exactly at this point of the fæculent abscess, there was this strange deformation;—the colon—the rectum—and the ileum: each, conjointly with the fæcal abscess, possessed one common entrance into the bladder itself.

Within the bladder, the rugæ of its mucous membrane were vascular, and its muscular coat was considerably hypertrophied.

### X. On Chorea. By B. G. Babington, M.D. F.R.S.

Dr. Babington remarks that,—previous to Dr. Hall's researches, the proximate cause of chorea was supposed to consist in debility, and some degree of irritation of the organic class of nerves; extending more or less to those of volition; and occasioning morbid susceptibility of the nervous system generally, with diminution of power, increased mobility, and irregular actions of the muscular system, particularly of those muscles supplied with the nerves principally affected.

Let us contrast this loose and general account of the pathology of chorea with that offered by Dr. Marshall Hall. He first maintains, as principles of physiology, that, besides the contractile power in muscular fibre itself, there are three causes of muscular motion:—1st, Volition; the seat of which is the cerebrum, and the action of which is conveyed along the fibres which decussate in the medulla oblongata. 2dly, The direct and reflex action of the excito-motory system. And 3dly, Emotion.—He affirms, that the seat of emotion is below that of volition; is in the medulla oblongata; and acts along fibres which probably do not decussate;—and, that the seat of the excito-motory system is in the spinal cord. He further remarks, that volition has an aim or object; while emotion, and the excito-motory function (or *vis nervosa* of Haller), are aimless on the part of the individual, and frequently opposed to volition.

According to Dr. Hall's earlier papers, chorea was considered an affection of the true spinal system; affording an example of the want of harmony between the cerebral and the true spinal acts. The volition was affirmed to be normal; but the true spinal act to be abnormal, for want of a precise harmony between the two. This view, however, did not account for the absence of chorea during sleep: for it is one characteristic of the excito-motory function, that it goes on during sleep; so that a disease asserted to be dependent on a morbid condition of that function, ought to be at least as manifest during sleep—when volition, as a disturbing cause, is abstracted—as in the waking state: but the contrary is notoriously the fact; all the symptoms of chorea ceasing as soon as the consciousness of the waking state is suspended. It was necessary, therefore, to seek further for an explanation of this circumstance. "It is well known," says Dr. M. Hall, "that the irregular movements in chorea, and in incipient paralysis agitans, subside during sleep. I was long perplexed to account for this fact. It was only by observing that these movements subside during quiet sleep only, and return during the agitation of dreaming, that I perceived that it is not sleep, but the absence of emotion, to which this effect is to be ascribed;—dreams during sleep having the same effect as emotion in our waking hours." This, then, I take to be his view of the pathology of chorea—that it is a morbid condition of the organ of emotion, which has its seat in the medulla oblongata, and is wholly independent either of the brain or the ganglionic system.

Dr. Babington adds:—

"I should define chorea to be a disease characterized by irregular uncontrollable contractions of the voluntary muscles, alternating with their atony, and occurring without pain. I have used the word 'contractions,' and have included the atony of the muscles in this definition, because the movements in this disease appear to me to differ essentially from those of convulsions and epilepsy in this,—that the stimulus, whatever be its nature, which excites either the whole or a portion of the voluntary muscles to involuntary action, is not more violent in degree than the normal stimulus of the will, or of the excito-motory system; so that movements, almost incessant indeed, but not exaggerated like spasms, are the result. It will illustrate my meaning, to state, that a person in sound health could, at any one moment, perfectly imitate, by an exercise of the will, every movement which he would involuntarily perform if he were the subject of the most aggravated form of pure chorea. Again, there is another circumstance which seems to me to attend the movements in chorea, and which may furnish ground of distinction between this and truly spasmodic seizures. The nerves, in their normal state, are always exercising a certain amount of influence over the muscles; so that where there is antagonism of forces, it is only necessary to remove the one opponent in order to demonstrate that the other is in a state of activity. This being the healthy condition, we have a right to consider the diminution of this activity as a morbid state; for although, from the striking effect which a morbid exaltation of muscular force produces, spasm is more directly brought to the cognisance of our senses than atony, still the latter is no less a really morbid condition than the former. I venture, then, to express my belief, that while, in true convulsions, the muscles, after having been thrown into a spasmodic state, do only return to the normal condition; in chorea, on the contrary, a further diminution of nervous influence occurs; so that the muscles become, in all marked cases, entirely passive and inert in the intervals between their irregular and involuntary actions. This is manifest, from the manner in which the limbs drop from the position into which they have been thus thrown; in which the head, after being tossed to and fro, will fall passively on the shoulders, and from the incapability on the part of the patient to hold any thing in his grasp."

Dr. Babington offers a good account of the history and symptoms of chorea. And his allusion to the exciting causes of the complaint is lucid. If we affirm, he says, that the primary seat of chorea is in the medulla oblongata and spinal marrow, we must at the same time admit that this may be affected through the medium of its connexion with the sensorium on the one hand, and with the ganglionic system on the other. Its exciting causes will thus naturally divide themselves into three kinds; namely, 1st, Those which primarily affect the spinal system: 2dly, Those which secondarily affect it through the sensorium: 3dly, Through the ganglionic system. Blows on the head or neck, causing a *contre-coup*, which shall either structurally injure or functionally disorder the medulla, occasionally give rise to chorea, not only at the time of their occurrence, but at uncertain periods afterwards: so also injury of the spinal marrow, by direct compression; alteration of its structure by rheumatism; and perhaps its irritation, by certain injurious practices;—the irritation of the incident nerves, by a wound, by the poison of mercury, of lead, of strichnine, and by skin diseases. Of those causes which act primarily on the sensorium, by far the most frequent is an affection of the mind, arising from any of the depressing passions; as from sudden fear, from horror, from grief. Perhaps, however, these causes should be rather considered as of the first kind, if the theory be adopted, that the seat of emotion is in the upper part of the spinal cord, and not in the brain. Other causes more evidently first affect the sensorium; as, organic diseases of the brain, fever, epilepsy, hysteria, and mental alienation. Of those causes which act primarily on the ganglionic system, may be enumerated, costiveness of bowels, with morbid accumulations in them, and worms of different kinds. Rheumatism also, when it affects the heart and pericardium, may give rise to the disease, through the irritation of the plexus and ganglia, which so entirely surround that organ, and the origin of its great vessels: and irregular menstruation may produce a like effect, through the lumbar plexus.

In a note we find the connexion between chorea and cardiac affections noticed still more pointedly. We transcribe the note in question.

"Out of a very large number of cases of chorea, seen lately by my friend and colleague, Dr. Addison, to whom I am indebted for having first directed my attention to this point, only two have been without a decided mitral or left ventricular bruit. In these two there was diseased heart; and in one case, examined after death, there was found old thickening of the mitral valve, with very recent pericarditis. Should further investigation prove chorea to be more immediately dependent on disease of the heart or pericardium, than has been hitherto supposed, the merit of the discovery will certainly be due to Dr. Addison." But we cannot help suspecting it will turn out otherwise.

Dr. Babington relates twenty-five cases, none of which require notice. The gist of the matter—the treatment, is thus handled.

He has not found any one remedy so superior in efficacy to the rest, as to induce him to abandon all others in its favour. On the contrary, the most powerful will sometimes disappoint our expectations; and we are then obliged to try one after another; and in the end, perhaps, remain uncertain, should the patient do well, whether the recovery is to be attributed to the means employed, or to the power of Nature herself. Dr. Babington, therefore, treats the cases *rationally*.

Where there is evidence of congestion in the head, marked by giddiness and headache, occurring in subjects of a full habit and florid countenance, the treatment should be commenced with moderate depletion; which, however, it would be more advisable to effect by leeches or cupping-glasses, than by the use of the lancet; and these should be applied to the nape of the neck, or behind the ears. Attention to the state of the bowels is of course, in all cases, indispensable, even though the general treatment should be of a tonic character: but wherever there is reason to suspect that the symptoms are dependent on a constipated or loaded state of the bowels, or their irritation from the existence of unwholesome aliment, purgatives should be administered freely and frequently; and those of the more active kind should be employed. As this state of the *primæ viæ* exists in a great many cases, it is not difficult to understand why the purgative plan of treatment has proved frequently successful. Where worms, and especially tænia, cause the irritation, turpentine, and other anthelmintics, will prove most successful; and these cases also will swell the list of those who will be benefitted by brisk and repeated purgatives.

Where there is reason to think that the disease is connected with the state of the uterus, occurring about the period when the catamenia should appear, and combined with symptoms of hysteria, those remedies will naturally suggest themselves which have a special power in causing this discharge, in obviating its irregularity, and in correcting its unhealthy character. The state of the teeth should also be looked to about the period of the second dentition; and even on the cutting of the dentes sapientiæ, as a probable source of irritation;—and the gums should be lanced, or the decayed roots of the first set removed, according to circumstances.

Where the the disease has arisen from a metastasis of rheumatism to the fibrous structure of the theca of the cord, it ought to be treated in the same way as pericarditis,—by depletion, general or local, antiphlogistics, and the employment of mercury, carried to slight salivation.

The following are Dr. Babington's opinion on the subject and comparative value of tonics.

" In a very numerous class of cases which owe their origin to sudden emotion, producing a strong impression upon a weak and excitable nervous system, the patient will be most benefited by all those remedies which improve the general health, and give vigour and tone to the nervous and muscular systems. The most severe case I ever saw recover, was cured in a few days by divided doses of port-wine, in which enough of sliced rhubarb was steeped to render it gently aperient. Various vegetable tonics have had their advocates; but bark and sulphate of quinine may be taken to represent them all. The metallic salts and oxides have, however, of late years, been generally preferred. Sesqui-oxide and sulphate of iron, sulphate of copper, oxide and sulphate of zinc, nitrate of silver, and arsenite of potassa, have all been tried, and found, in different hands, to succeed. The testimonies in favour of sesqui-oxide of iron in large doses, and of sulphate of zinc, are perhaps the strongest. On the latter remedy I have generally, in the cases which I have just alluded to, most relied: and my expectations regarding its efficacy have seldom been disappointed. I have found it necessary to administer much larger doses, however, than are usually given; good effects seldom being perceptible until twelve or fourteen grains are taken three times a-day. By gradually increasing the quantity a single grain at a time, even much larger doses than this may generally be employed, without exciting sickness, and with the best effect. I have known half-drachm doses, thrice a day, taken for several weeks in succession.

Sulphate of zinc, however, will not be borne by all stomachs, even in small doses; and we are then obliged to give up its employment, long before we have attained even the minimum dose requisite to give it a fair chance of controlling the disease. In such cases, I generally have recourse to the liquor potassæ arsenitis, cautiously increased in its dose from three to twelve or fifteen minims, according to the age and strength of the patient, and other concomitant circumstances. I believe this is the most powerful remedy of all—at least I have found it so, in several obstinate cases; but I am deterred from employing it, where other remedies will succeed, from the sickness and griping pains which it is apt to cause, and from some fear that the constitution may be permanently injured by its continued employment.

As an external remedy, the shower-bath may be very often advantageously used, in conjunction with internal means; and I have even tested its efficacy with success, when used alone. In St. Petersburg, I am informed by a Russian physician, a new practice has, within the last year, been adopted with eminent success in obstinate cases of chorea. The patient is placed in a bath as hot as he can bear it; kept there for half-an-hour; and, when thus thrown into the most profuse perspiration, is suddenly plunged into cold water.—I have not ventured to try this method of producing a sudden shock; or rather, I should say that opportunity has been wanting, since I have been made acquainted with it; but in an extreme case, and when other remedies had failed, I should, on the testimony I have received in its favour, not hesitate to employ it.

The treatment by electricity is very advantageously revived at Guy's Hospital. We have given an account of Dr. Golding Bird's paper upon this subject.

---

### BALTIMORE HOSPITAL.

Report of Cases treated in the Hospital. By Samuel Annan, M.D. Senior Physician to the Institution.*

From amongst the cases reported by Dr. Anann, we shall select one or two.

Case 1.—*Paralysis of the Left Side of the Trunk and Extremities, and of the Right Side of the Face.*—S. G., ætat. 28, negro, admitted April 7th, died June 5, 1840. On the 14th of May, 1839, while engaged in washing, was suddenly seized with an acute pain of the right side of the head, and fell down in a state of insensibility, in which state she remained during twenty-four hours. When she recovered her senses, she found she had entirely lost the power of moving her left arm, and in a great degree that of moving the leg of the same side. The right side was unaffected, with the exception of the face, the muscles of which had become paralyzed; those of the left side of the face still retained their accustomed power of motion; when her tongue was thrust out, it inclined very much to the right side; the sensibility of the left side was destroyed, and likewise that of the right side of the face; she could not hear with the right ear. The right eye became inflamed several weeks before her death, and the cornea was slightly ulcerated; the upper eyelid was constantly raised. When she attempted to speak her muttering was scarcely intelligible; paralysis of all the parts affected was complete; deglutition and mastication were performed with great difficulty.

*Dissection twelve hours after Death. Brain.*—There was venous congestion of the surface, and of the medullary centres of the hemispheres of the cerebrum. There was a fibrous, semi-cartilaginous tumor on the right side of the tuber annulare and medulla oblongata, seated in the substance of the dura mater, arachnoid membranes and the pia mater. It extended from the point where the fifth pair of nerves arises from the tuber annulare, covered the origin of this nerve and the whole of the right side of the tuber below this, and passed down along two-thirds of the medulla oblongata, and adhered to the right side of the basilar artery. The right vertebral artery was enclosed in the substance of the tumor. It was about two inches long. The surface of the root of the right crus cerebelli, on which it pressed, was softened, as was also that part of the tuber annulare, on which it lay. It was incorporated with the substance of the right side of the medulla oblongata, and had produced softening as far as it reached. This softening extended through the posterior tract, but became less as it approached the posterior surface. The anterior tract was a pulpy mass. Neither the anterior nor the posterior tract of the left side was perceptibly affected. The tumor pressed upon, and had caused softening of the roots of the fifth, seventh, eighth and ninth pairs of nerves. The bone was rough about the foramen lacerum posterius, and the condyloideum anterius. The liver was of a yellow colour.

---

* American Jounal, Med. Sciences, July, 1841.

The remarks of Dr. Annan are physiologically just and instructive. "The symptoms," he observes, "in the case given above, corresponded very exactly with the seat and extent of the disease. The right side of the medulla oblongata was softened to the extent of complete disorganization. There was complete paralysis of both motion and sensation on the left side. The decussation of the fibres of the corpora pyramidalia, explains the loss of motion of the opposite side; but as we have no facts proving a similar interlacement of the fibres of the posterior or sensory tract, it is not so easy to discover how it happened that the right side was not deprived of sensation. Motion and sensation were unimpaired in the extremities of the side diseased; they were both destroyed in the same parts of the opposite or left side. Are we not justified, from this, in making the inference, that there is a decussation of the filaments of sensation, as well as those of motion?

The fifth pair of nerves is formed, on each side, of two sets of filaments, viz., one for motion, the other for sensation. The former take their origin from the intercerebral commissure, which lies between the cerebrum and cerebellum, and is composed, in part, of the valve of Vieussens; while the latter can be traced down the posterior columns of the spinal cord, about an inch and a half below the tuber annulare. They were both pressed upon, by the upper part of the tumor, at the point of their emergence from the tuber, and the sensory portion, was involved in the general destruction of the texture of the medulla oblongata. The motory filaments being distributed to the muscles concerned in the motions of mastication, viz., the masseter, temporal, pterygoid, and buccinator, and those of the right side being paralysed, the power of masticating food was consequently rendered imperfect. The sensory portion, distributing its filaments to the mucous membrane of the nose, of the palate, the pulpy structure of the teeth in both jaws, the papillæ of the tongue, many parts contained within the orbit, the lachrymal apparatus, the conjunctiva, &c. and the skin covering the face, all these parts were necessarily deprived of sensibility. The seventh pair, formed under the old division of the facial and auditory nerves, or of the portio dura and portio mollis, is attached to the medulla oblongata, between the corpus pyramidale and olivare, just below the pons Varolii, and was involved in the disorganised mass. The facial nerve supplies the muscles of the face, including the orbicularis palpebrarum. The paralysis of this muscle prevented the closure of the eyelids; and the consequent exposure of the eye to particles of dust contained in the air, with the diminished sensibility of the conjunctiva, from the paralysis of the sensory portion of the fifth pair, the eye not feeling the irritation, of course no tears were secreted to wash out the irritating matter, explains the inflammation of the conjunctiva with ulceration of the cornea. The auditory nerve being destroyed, there was deafness of that ear. The glosso-pharyngeal, par vagum, and spinal accessory nerves, were all destroyed. The first supplies the muscles of the pharynx and tongue: the second distributes motor filaments to the larynx, furnishing the muscles concerned in the production of vocal sounds with motory power. The ninth, or lingual nerve, was likewise included in the disorganised mass. It goes to the muscles of the tongue, and also to those of the os hyoides. The muscles of the larynx and tongue of one side, having thus lost their power of motion, speech and deglutition were rendered imperfect. The genio-hyo-glossus of the opposite side, retaining its power of acting under the will, pushed the tongue to the right side when it was thrust out. We thus have a satisfactory explanation of the whole extent of the paralytic affection."

CASE 2.—*Pleuræ Pneumonia.—Pneumo-Thorax.—Gangrene of the Lungs.*—M. R. ætat. 19, admitted May 2nd—died May 3rd, 1840. This girl was a prostitute, and the persons who brought her to the institution, said she had been ill two weeks; she was delirious, and incapable of giving any account of herself; she had been attended by a physician, but no information as to the treatment adopted could be procured; she was very restless, and was constantly talking and groaning: the skin and white of the eyes were of a bright yellow colour; tongue dry, and coated with a yellowish brown fur; teeth covered with dark brown sordes; respiration frequent, but not laborious; pulse 120, and of tolerable strength: there was general dulness of the chest, on percussion, with feeble respiration. Eight ounces of blood were taken from her arm, and she was ordered two grains of calomel every two hours until it purged. Early the next morning, her breathing suddenly became very laborious, and in the afternoon was extremely so, accompanied by great heaving of the chest; pulse 120, full, but compressible. She was ordered infusion of serpentaria, with camphor julep; she died that night.

*Dissection*, 12 *hours after death.*—*Brain.*—There was great congestion of the large veins of the surface of the hemispheres, and numerous, and large red points were visible, on slicing the medullary substance. *Thorax.*—On cutting into the left cavity of the chest, air rushed out with a whizzing noise. The left lung was pressed close to the spine, and was of a dark brown, and in spots, of a black colour, and had a coating of dark brown lymph, tinged with yellow, over nearly its whole surface. Eight or ten spots, some as large as a walnut, were in a state of sphacelus; and were reduced to a black, soft, pulpy mass. These, when cut into, gave out a very fetid gas. The pleura had given way over one of them, and air had escaped; the larger part of this lung was hepatized. The bronchial tubes were of a dark red colour. The right lung presented the same appearances, but in a less degree. Only two or three spots were gangrenous, and hepatization had not advanced so far. *Abdomen.*—The mucous coat of the stomach was considerably congested. That of the small intestines in a slight degree; the other viscera were normal.

Dr. Annan remarks:—"This case which I have just narrated, is remarkable for the extent of the disease, and the occurrence of hepatization between the gangrenous spots. Both lungs would appear to have been very generally affected with pneumonic inflammation, which being improperly treated, and the constitution having been greatly debilitated by previous excesses, it passed rapidly into gangrene. Hepatization took place over all the spaces between the gangrenous spots. If the vital powers had not been so greatly impaired by a long course of dissipation, the pneumonia would have passed on to its ordinary third stage, viz. purulent infiltration. There would appear not to have been sufficient energy to elaborate pus. It is surprising, too, that there was neither cough nor expectoration. The delirium was the prominent symptom, which made me suppose that the brain was the organ chiefly implicated. Dissection showed congestion of the brain but not in sufficient degree to have proved fatal. If, instead of exhibiting incoherent muttering and groaning as the prominent symptoms, she had been harassed with incessant cough, accompanied by fætid expectoration, the case would have been plain enough. As it was, the attention was drawn off from the lungs to the brain. It should, however, be remembered, that fætor of the sputa alone, is not pathognomic of gangrene of the lungs. It is true, that the breath and sputa, are, in this disease, from the first, nearly as offensive as when the fætor becomes of the true gangrenous character. This is owing to the depraved condition both of the fluids and solids. But it is well known, that this state of things often occurs where gangrene of the lungs does not exist." No doubt too great importance has been assigned to fætor of the sputa as a characteristic sign of gangrene of the lungs. But if, in addition to this fætor, the sputa are observed to be bloody, brownish, or greenish—something like the discharge from a sloughing part; and when any thing like green fragments of lymph are seen, together with a weak pulse, an elongated countenance, and a cadaverous aspect; in short, when the patient is in the state in which we see people when they are sinking from mortification of any other part; there can be no doubt of the nature of the disease.

### GLASGOW ROYAL INFIRMARY.

CASES WITH OBSERVATIONS. By WILLIAM DAVIDSON, M.D. Senior Physician to the Infirmary.*

1. The first case detailed by Dr. Davidson is one of chronic pleurisy. Paracentesis was performed between the 6th and 7th ribs on the left side. Twenty pounds (apoth. weight) of serum were evacuated. It had a yellowish colour, was nearly transparent, adhesive, almost solidified by heat and nitric acid, and had a specific gravity of 1016. The fluid re-accumulated, and a small grooved needle was inserted into the left pleural cavity, near the cicatrix of the former opening, and, a large cupping-glass being applied over it, forty ounces of reddish-coloured serum were drawn off. Next day a small trocar was inserted by Dr. Lawrie, and twelve pounds (apoth. wt.) of serum were drawn off by cupping-glasses. The patient subsequently died.

Dr. Davidson makes some remarks on the mode of performing the operation. "The operation for empyema is not very frequently performed, for on the one hand there are the difficulties of the diagnosis, and even when the signs are pretty clear, there may be some doubt about the state of the lung; and on the other, the danger of exciting a pleuritic inflammation. A plan, therefore, which may tend to remove or lessen any of these objections is worthy of consideration. The chief danger of the operation arises from the admission of air into the pleural cavity, and this cannot be avoided by the ordinary method, and if the stethoscope be applied to the chest during the evacuation of the fluid, atmospheric air can be heard entering its cavity with a noise similar to that produced by emptying a bottle nearly filled with water. To obviate this result, it occurred to me that the fluid might flow through the channel of a grooved needle, with the exhausted cupping-glass placed over it; this I accordingly tried, as stated in the report, and it succeeded perfectly, but the quantity of fluid being very great, it was necessary, in the second trial, to use a small trocar. One practical difficulty occurred with the canula, but not with the grooved needle, viz. its liability to slip from the opening, when the cupping-glass was applied, which is obviated by previously tying it around the chest by a piece of narrow and very thin ribbon. The cupping-glass employed was curved, and capable of containing about two pounds of fluid, and it was exhausted by a piece of ignited lint, which had previously been dipped in alcohol. Very little air, as far as could be ascertained, entered the pleural cavity, and it is a good practical rule to hold the finger over the mouth of the canula, during the changing of the glasses, which ought to be frequently done, as it lessens the duration of the operation, by causing the fluid to flow in a full stream, whereas, without the aid of an exhausted cupping-glass, it would do so very slowly. The same plan of operation I have frequently adopted in opening chronic abscesses, even in the knee-joint, without any subsequent irritative fever or inflammation, and in one or two cases, even in cachectic constitutions, there has been no re-accumulation of matter, when firm bandaging was afterwards had recourse to."

In another case, the operation was conducted exactly as described in the former, and the narrow piece of ribbon employed was completely successful in

* London and Edinburgh Journal of Medical Science, No. 11.

retaining the canula. A few bubbles of air got admission into the pleura, which is inferred, as stated in the report, from the metallic tinkle, which was heard two or three times. This may, he thinks, be obviated, by withdrawing the cupping-glass whenever the fluid ceases to flow in a stream, and applying another one.

2. *Rubbing Sound in Pericarditis.*

W. A., aged 15, of delicate and rather unhealthy constitution, was seized, on the 10th April 1841, with a slight shivering, some pain in left side of chest, slight dyspnœa, cough accompanied with a slightly accelerated pulse, hot skin, and furred tongue. On the following day the chest was examined, but nothing was discovered abnormal, except a pretty general bronchitis. On the 16th, he complained of rather acute pain in the cardiac region, accompanied with dyspnœa, the pulse being 100, moderate in strength and regular. On percussion, the whole of anterior chest was found clear, except the right submammary region, which was slightly dull, and a dry subcrepitation was heard there with the stethoscope. The sounds of heart were rather loud, the impulse being pretty strong, and a rubbing sound was heard accompanying its action, about an inch below nipple, and a little to its right. This rubbing sound was tolerably distinct even during respiration; but in order to be sure that it was not connected with this process, the patient was desired to hold his breath, when it was most distinctly recognised as accompanying the motions of the heart. Although I listened to it very attentively on several occasions during the three or four days that it was discoverable, I was unable to connect its precise similarity to any other friction sound with which the generality of men are familiar. It certainly was not like the creaking of leather, but if I were obliged to give an expression to my perceptions, I would say that it resembled somewhat the sound produced by the friction of one piece of woollen cloth against another. This friction sound, as already mentioned, only continued for a few days, and after that period, there was distinct and extended dulness of percussion in the cardiac region, accompanied with palpitation, irregular pulse varying from 120 to 130. On the 6th May, his symptoms were the following—urgent dyspnœa; palpitation on the slightest movement; pulse 130, irregular, intermittent, weak; frequent cough; expectoration—a greenish, tenacious mucus; dulness of percussion in cardiac and both submammary regions; action of heart, weak, irregular; dry subcrepitation in right submammary, and in part of left submammary regions, accompanied with considerable fulness in the intercostal spaces of left chest and epigastrium, with œdema of the lower extremities. He lived nine days after this period, but the symptoms did not differ materially from those now mentioned. His treatment consisted of antimonials, purgatives, calomel and opium to salivation, leeches to the cardiac region, repeated vesicatories, diuretics, and, towards the close of his disease, anodynes.

*Inspection.*—Inferior lobes of both lungs were red, congested, and slightly crepitant, bronchial tubes were reddened, and they contained an opaque somewhat tenacious mucus.

Pericardium was enormously enlarged, extended beyond sternum a considerable way towards right side, and covered completely the lower half of left lung. On laying it open, about three pounds of thin whitish pus, mixed with white-coloured flakes of lymph, were evacuated. The internal surface of pericardium was whitish, rough, and covered with numerous patches of lymph and adherent flocculi. The heart was large, very flaccid, rather displaced or twisted to the right side, to which its apex pointed almost directly. Its pericardial covering was almost completely coated with patches of lymph, and rough fibrous vegetations. The valves and internal membranes of heart appeared to be quite normal. No other morbid appearance was discovered in the chest or abdomen. The head was not examined.

3. *Diagnostic Symptoms of Malignant Disease of the Liver.*

The symptoms of the disease during life are involved in still greater obscurity than its pathology, and, in the early stages, our opinions must be little better than conjectural. The general symptoms of gastric and hepatic derangement are not to be depended on, for they are common in other affections of the liver; and Cruveilhier states, that the enlargement of this organ, and the elevations on its surface, are the only pathognomonic signs. In the majority of cases that I have witnessed, pain, frequently of a severe kind, was experienced in the right hypochondrium or epigastrium, and the termination of the disease was more rapid than in other hepatic affections. If, then, the liver be found considerably enlarged, distinctly nodulated—if the disease be accompanied with severe pain in the hepatic or epigastric regions—if it be somewhat rapid in its progress, and if the features of the patient have a very cachectic or sunken appearance; then there can remain very little doubt of the malignant nature of the affection.

---

### CLINICAL REMARKS BY DR. MARSHALL HALL, ON THE USE OF SETONS.*

Many years ago, I was consulted by Mr. Doubleday, of Blackfriars Road, in the case of a young married lady, who had suffered from peritonitis after her first accouchement.

This peritonitis appeared to be confined to the pelvic region. Its acute character had been subdued, but tenderness, with tumidity, and difficulty in voiding the bladder and rectum remained. I made a careful examination. A distinct hardness was felt under the pubes, extending to one side, I think the left. On examination per vaginam and per rectum, a similar hardness was found occupying the lower part of the pelvis. I imagined this hardness to consist in coagulable lymph, effused from the inflamed peritoneal surfaces of the pelvis, producing the symptoms by its pressure on the neck of the bladder, and on the rectum.

We strictly regulated the diet and the intestines, and inserted an ample seton over the induration. Slowly and gradually that induration, with its attendant symptoms, became diminished, and eventually disappeared.

Several years after this, I was consulted in the case of the sister-in-law of this patient, under very nearly similar circumstances. The same remedy was followed by the same happy result.

A year ago, I was consulted by Mr. Burford, in the case of a gentleman of sixty, who had become affected with pain, tenderness, and tumidity of the abdomen. On a careful examination, a distinct hardness was felt, in the midst of the general tumidity, occupying the region of the caput cœcum coli. We regulated the diet and the bowels, administered mercury, and inserted an ample seton. The mouth became affected, and the seton discharged copiously: the hardness and the other symptoms gradually, but at length, entirely disappeared.

A similar case occurred a year ago, in the person of a gentleman of forty, a patient of Mr. Squibb, in Orchard Street. A strict regimen was enjoined, the bowels regulated, and an ample seton was inserted. The induration, which in this case occupied the space between the false ribs and the ilium, on the left side, gradually disappeared.

Two years ago, I was consulted by Mr. P——, a barrister, affected with pneumonia of the middle and upper lobes of the right lung. A seton was in-

* London and Edinburgh Journal of Med. Science, No. XI.

serted, and Mr. P—— went to Madeira. On his return, the physical signs and the symptoms of the pneumonia had disappeared.

I have still more recently treated a case of pneumonia of the upper portion of the right lung, in consultation with Mr. Beane of Peckham. A seton was inserted, and in six weeks a most decided amendment in the physical signs, the symptoms, and the general health occurred. Since that period, the patient has continued to improve, and now no dulness on percussion, or other sign of disease, is perceptible.

In a variety of cases of acute or chronic, local or limited internal inflammation, I have had recourse to the seton, and uniformly with the most marked success; so that, I think, we may look upon the remedy as almost specific in such cases. It is unnecessary to enumerate them. But hepatitis and nephritis belong to them in an especial manner, and I would suggest this remedy as likely to be of service, (if any remedy can,) in the case of albuminous urine. In one such case the urine was more albuminous after cupping. I imagined the effect arose from the mechanical violence inflicted, and recommended the cupping to be performed above and below the precise region of the kidneys. Under the use of this remedy, the albumen diminished, and even ceased for a time.

These and other cases, then, induce me to think, that there can be little doubt of the real efficacy of the seton in chronic inflammation. The object is to demonstrate this in some measure, and then to notice briefly, farther applications of the remedy. I do not pretend to suggest anything new, but rather to enforce what is old. The efficacy of setons, when appropriately applied in the nucha, (for they are frequently employed very uselessly,) is well known. The proper cases are inflammation and congestion. But the case to which I would particularly draw attention is that of disease of the spinal marrow, with paraplegia, or paraplegic spasm.

In this case, issues are generally inserted. They appear to me far more painful, far less manageable, and far less efficacious than ample setons. They have also, I am persuaded, been generally applied *below* the real seat of the disease. I was consulted a few weeks ago by a gentleman from Manchester. With partial loss of power, he had loss of sensation in the lower extremities; the numbness extended to a line just above the sacrum. Issues had been applied on each side of this line. They might, with equal efficacy, have been applied to the foot! I need not say that the spinal nerves proceed, for some distance, from above directly, rather than obliquely, downwards, and that the seat of the disease is *at* or ABOVE their junction (insertion or origin,) with the spinal marrow.

Bearing these two principles in mind, then, viz. that ample setons afford a more efficacious counter-irritation than issues, and that they ought to be applied higher along the spinal column than has been usual, I think we have a new mode of treatment for this formidable class of diseases.

These setons should, besides, be larger than usual. They should be three-fourths of an inch in breadth, and extend through two inches in length, be inserted on the level with and above the supposed seat of the disease, (the anatomy being consulted,) and be four or six in number, two or three being instituted on each side of the spinal column. Acting on this principle, I had, five days ago, the pleasure to receive the most satisfactory account of a patient affected with paraplegia, whom I had seen at Lohan, in Essex, in consultation with Mr. Gross.

I repeat, and beg to conclude by repeating, that I believe counter-irritation applied along the spine has failed, because it has been applied below the seat of the disease; and that, to be efficacious, it must be both more efficient in itself, and applied with greater regard to the anatomy of the spinal marrow and nerves. The precise spot for their application must be left to the well-informed practitioner. I need scarcely remind my reader, that the persistence, or cessation, of all reflex actions, will determine whether our remedies should be applied above or below the origin of the cauda equina, above or below the last dorsal vertebra.

We are great advocates for setons too. We confess we have not used them so broad nor so many at a time as our friend Dr. Hall. But perhaps he is right. The difficulty would be, in some cases, to induce patients to submit to their introduction.

We have tried setons in three or four cases of albuminous urine. They were bad cases certainly, and the effect was not encouraging.

---

### MR. WILDE ON THE SCHOOL OF OPHTHALMIC SURGERY IN VIENNA.

Mr. Wilde gives an interesting sketch of this celebrated School in the last number of our Dublin contemporary.* We shall extract some passages from it.

*State of Surgery in Austria.*—It is remarkable, that while ophthalmology is, and has, for so many years been cultivated with such marked success in Austria, the general practice of surgery is in a state so low, that one of the grades of those licensed by its universities and lyceums to practise that branch of the healing art, is compelled by law to keep a barber's shop, whose interior may be learned by a glance at Teniers' graphic illustration of a Dutch surgery.

*Barth the first Teacher of Ophthalmology in Austria.*—Barth was born in the island of Malta, in 1745, and studied medicine at Rome, and afterwards at Vienna. When but eighteen years of age, he was appointed Professor of Anatomy to the University under Stöerk, the successor of Van Swieten. The anatomical school of the Austrian capital acquired considerable renown at that period, from possessing the valuable microscopic preparations of Ruysch, Lieberkühn, and Albinus, purchased by Van Swieten for the University; they were committed to the keeping of Barth, and the opportunities they afforded him for studying minute anatomical structure were eagerly laid hold of, and tended in no little degree to his future advancement.*

This tradition is current in Vienna; a lady attached to the court of the empress becoming blind, was pronounced amaurotic by the medical advice called in; her malady continuing to increase, the Baron Wenzel was sent for, and at once declared it to be cataract; and operated on it with success. So amazed was Maria Theresia, at this display of Austrian surgery, that she forthwith established a special lectureship of ophthalmology, and Joseph Barth was the first that filled this chair, in 1773; and in 1776 he was appointed oculist to Joseph the Second. He was a most expert extractor, and there are still living several who have witnessed his operations—the invention and use of Beer's knife (that now so generally adopted) is in a great measure due to him: for although his was longer in the blade, and somewhat broader toward the handle, yet it was upon an enlarged scale the same. The objections to it, of its pricking the nose from the great length of its point, and its not cutting itself out (as it is termed) with

---

* Dub. Journ. Nov. 1841.

* Several of these most beautiful preparations still remain in the University museum; those of Luberkühn in particular, now in the keeping of Professor Berris, are, notwithstanding all our modern improvements, some of the finest injections in existence; they are only equalled by those of our esteemed friend, Professor Hyrtl, of Prague.

facility, is now obviated in that introuced by his pupil Beer. His mode of operating was remarkable; he did not require an assistant, and (was perhaps the first who did so), but placing the patient standing in the corner of the room near a window, he opened the lids, and fixed the eye with one hand, while he passed his knife through the cornea with the other, as is now so dexterously performed by Mr. Alexander; but different from that most distinguished oculist he stood *before* his patient.* It is needless to add that he was ambi-dextered. Barth wrote upon anatomy, and published sixty-one plates of the muscles, in Vienna, 1786. His operations are to be found in the works cited below. He also wrote a small treatise on cataract, (*Alhandlung über die Ausziehung des grauen Staars,*) in 1797. It is strange, that none of the writings of Barth are enumerated in Engelman's catalogue. He died in 1818, his portrait bespeaks him a man of noble and prepossessing appearance, and his *ad captandum*, but engaging manner and address, added to his acknowledged talents, procured him many admirers.

*Santerelli, the Author of the Upper Section of the Cornea.*—To Jacob Santerelli is undoubtedly due the first performance of extraction through the upper section of the cornea. Dr. Mackenzie says, that when he was a pupil at Vienna, in 1817, " it was usual to attribute the invention of the upper section to Santerelli, and to swear in *verbo magistri* that it was a bad operation." It has been frequently claimed by others, we believe unjustly. Dr. Mackenzie continues, " Santerelli was the first (Delle Cateratte, p. 79; Forli, 1811,) as far as I know, who actually made the section, not semilaterally as Wenzel had done, but at the upper edge of the cornea. This he did at Berlin, in 1795." The method of Santerelli was to operate standing behind the patient, who was seated beneath him, similar to that of Mr. Alexander. He opened the cornea, by inserting a knife, shaped like a broad-shouldered lancet, into the anterior chamber, through its upper edge, acting in this manner like a wedge, and not giving the clean incision made by dividing it from side to side. Dr. Mackenzie doubts, (and it appears to us with great justice,) whether he could in this way divide more than a quarter, or, at most, a third of the circle of the cornea; yet both Rosas and Jünken state, that he fairly opened the upper half of the cornea. The latter of these authors adds, that he latterly abandoned this method, and made the under incision.

*Jäger's Clinique.*—Dr. Frederick Jäger, the son of a physician of Mergentheim, in Wurtenburg, was the favourite pupil, and afterwards the assistant of Beer. He resided in Vienna in the former capacity in 1808, and on taking his Doctor's degree at Landshut,† in the same year, he wrote an inaugural dissertation on " The Diagnosis of Arthritic and Syphilitic Inflammation of the Eye." From 1808 to 1812 he continued the assistant of the great Austrian oculist, who speaks thus of him in the little work already quoted, *Geschicte der Augenheitkunde*; " Since then," *i. e.* from 1808 to 1812, " he was uninterruptedly my assistant, and so advantageously distinguished himself by his diligent application, that he not only (under my direction) undertook, in private, the extraction of a cataract, but also publicly, in the clinical school, operated successfully by means of the same operation on both eyes of John Haas, a man aged 55, on the 19th of June, 1812." In the same year, Jäger published his " *Dissertatio de Karatonyxidis Usu,*" in which he records the results, and descants upon the merits of nineteen operations for cataract by keratonixis. On the death of J. A.

---

* Ehrlich, Chirurg. Beobachtungen. Th. 1. S. 34—und Saltz medic. Zeitung. Jahr, 1797. B. 2, S. 33. The first of these was published at Leipzig in 1795.

† The university of Landshut was transferred to Munich in 1826.

Schmidt, Dr. F. Jäger was appointed special Professor of Ophthalmology to the Josephinium Academy, a place he still continues to hold; and, as an operator has obtained nearly the same exalted reputation enjoyed by his master; and his private teaching is at present one of the greatest attractions in Vienna.

His clinique is on the same plan as that of the Grand Civil Hospital; it contains two wards, with eleven male and eleven female beds; the students are those educating for medical officers of the Austrian army; and the patients, soldiers and their families. Connected with this is a large *ambulatorium*, or dispensary, for out patients, or indeed all who choose to come; and around the Professor's chair will be found medical men of nearly every country in Europe, as well as America, attracted by the splendour of his operations, and attached to him by the unvariable kindness, and winning urbanity of his manner. The business is conducted on the general principle of the other German cliniques; it commences at eleven and ends at one o'clock. Between the wards is a spacious hall; into this the patient who is to be examined is conducted by a pupil, called *ordinarius*, in whose charge he is placed, who first gives the history of the case (in German,) and then proceeds with the subjective symptoms, and lastly, the description of the present appearances. He is then questioned on the case by the Professor, and concludes with the diagnosis, prognosis, and therapea. The Professor then generally makes some observations on the peculiarity of each example of disease, as it presents itself in the persons so examined.

The second hour is usually occupied with operations. Of Jäger's modes of operating, and the principles of his treatment, we shall speak hereafter. On the whole, we may say, that the latter is very similar to that pursued in the London Ophthalmic Hospital. Finally, those patients not able to be removed from their beds, are visited.

Occasionally the Professor holds a public examination of all the pupils in his class, and his assistant gives a course of public lectures on the operative surgery of the eye, twice a week.

*Ophthalmic Diseases in Vienna.*—Cataract and amaurosis are diseases of very frequent occurrence in Vienna, as also arthritic affections of the eyes, particularly arthritic iritis, whereas the syphilitic form of that disease is very rare in comparison with other countries, but especially in Great Britain, only three cases of it having presented in the Josephinum Eye Clinique in the last year.*

During the warm season, severe ophthalmias predominate more than with us, and many of them run rapidly into the purulent, or even the Egyptian form, particularly among the soldiery. The hot winds laden with quantities of fine dust, very similar to that in Egypt and other parts of the Levant, which prevail during the summer months, and are so annoying upon the Glacis and in the Vorstadt of this great city, are, no doubt, a fruitful source of ophthalmia to the Viennese. Chronic keratitis, the *pannus* of continental writers, is also very common here, and has received of late years much attention from the German oculists.

*Inoculation of Purulent Ophthalmia for Pannus.*—The treatment of pannus, or chronic corneitis, by producing a new inflammation through inoculation with the matter of *ophthalmia neonatorum*, though almost unknown in these countries, has

---

* There are several hundred persons treated in the syphilitic wards of the great hospital of Vienna yearly, both male and female, with every variety of venereal disease *without* mercury; and so rarely and sparingly is this remedy employed in private practice, that it can be fairly said the Austrian treatment of this malady is *non mercurial;* yet secondary affections, particularly those of the eye, are fewer than in other countries.

been employed for many years in Germany; and has lately attracted particular attention from its mention and recommendation in the work of Dr. J. F. Peringer, oculist to the hospital at Grätz, "*Die Blennorrhœ am Menschenauge;*" and in particular in the chapter "*Die Heiling des Pannus durch Einimpfung der Ophthalmoblennorrhœ.*"

Prior to the appearance of this very laborious work, so admirable in its symptomatology, our attention was directed to the subject by Professor Jäger, in whose clinique we had an opportunity of observing two cases so treated in November last.

The cases are related but do not appear to us to be particularly tempting. We rather think the practice would run counter to our habits in this country.

*Clinique of Rosas.*—As this clinique has been already described in the days of its venerable founder, a brief notice of it will here suffice. It consists of a well-arranged auditorium, where lectures are delivered and operations performed, and two wards, containing twenty beds, most admirably fitted up for the comfort of persons labouring under eye diseases. The annual reception of patients into this clinique is about 150, all interesting cases, chosen by the Professor's assistant, from the wards of the entire hospital, which contains 2,500 patients, and occupies the largest space of ground of any such institution in Europe. With this clinique is also united an ambulatorium, which affords relief to above one thousand persons yearly. The only difference in the routine of business here is, that the language spoken is Latin, and the *ordinarius* proceeds first with the objective, and then with the subjective symptoms; and immediately before each operation he reads aloud a Latin dissertation upon the case, its history, the objects of the operation, and probable result. On the whole, it is more methodical than that in the Josephinum, and perhaps this, added to the pains taken to instruct in the general treatment of patients, make this establishment better adapted to the improvement of students, whereas that of Jäger is better suited to the already initiated, and the more advanced oculist. After the ordinary duties of the clinique in the theatre are ended, the Professor visits those unable to be removed from their ward, where most valuable information can be gleaned from his observations. Without drawing invidious distinctions between the relative merits of these two great men, it may, perhaps, be near the truth to say, that while the operations of Jäger are the most splendid exhibitions of eye surgery in Europe, the therapea (treatment) of Rosas is superior; and, as far as the history of his science goes, the latter is said to be the more learned of the two.

*Plan of Study in Vienna.*—The most advisable course to be pursued by those who visit Vienna solely for the sake of the eye practice, is to attend Rosas, from ten to half-past eleven; then a few minutes' walk will conduct one to the school of Jäger, where he should remain till half-past twelve or one. From one to two Jäger has a public *ordination,* as it is termed, at his own house, for the reception of patients from among the middling classes, to which he generally invites strangers, but to which his private pupils always have access. Much can be learned here of his private practice; and no one visiting Vienna but should, if possible, get access to it. We now arrive at the most attractive portion of the course, and by far the most valuable part of the education given in eye surgery —the private course of instruction in operating. This occupies the hour from two to three, daily, at the Professor's house, when eyes can be procured, and for this a stated sum is required. He takes but six pupils at a time, and these are almost invariably foreigners, either private individuals, or persons sent by their governments or their universities. This course lasts generally about three months. Of the advantages it offers, as well as Jäger's and Rosas' modes of operating, we purpose to devote another chapter. Enough is it for the present to say, that there is no such inducement held out to visit any other continental school: and of its teacher we may justly say, in the words of a late writer on the subject, "*und was Allen bekant ist, mag genügen, dess Niemand seinen Unterricht ohne Befriedigug genossen hat.*"

Private courses on operative ophthalmology are likewise given occasionally by Dr. Rosas, and by the assistants of both Professors.

That of Dr. Gubz, the assistant in the general hospital, should not be neglected, as he gives some most interesting information on the history of his art, illustrative of the splendid collection of instruments.

### PERTH INFIRMARY.

### DIFFICULTIES IN THE DIAGNOSIS OF TUMORS.*

Dr. Thomson, surgeon to the Infirmary, has communicated some cases illustrative of the difficulties of diagnosis of tumors, more especially of those of the abdomen.

CASE 1.—*Scirrhus of the Transverse Arch of the Colon simulating Fæcal Accumulation.*—David Whyte, aged 60, carpenter, admitted March 4, 1840. Has for some time complained of severe pain situated at the umbilicus, occurring in paroxysms, and said to be of a twitching kind. Bowels always costive, stools dark and lumpy, generally finds relief from purgatives. There is a distinct tumor felt in the abdomen a little above the umbilicus, in the situation of the transverse arch of the colon, crossing to the left side, where it disappears under the ribs. It is quite moveable, and can bear examination without much pain. It seems to be composed of several round bodies in a line. He first began to complain about four months ago, and ascribes his complaint to a cart passing over his abdomen about a year previous.

As he always felt relief after purgatives, these were frequently given at his own desire. Not long, however, after his admission, the stomach refused to retain either food or medicine, and injections, which from the first were frequently administered, were finally abandoned, from the impossibility of making them pass up the gut; an examination of the rectum threw no light on the subject. A few days before death constant vomiting took place,—he died a month after entering the hospital.

*Inspection, 24 hours after death.*—The transverse arch of the colon was the seat of a large scirrhous mass, about five inches long and one thick, divided by fibrous bands in distinct portions. The whole of the intestines partook of the same diseased condition. The diameter of the larger bowels was diminished to the size of the little finger, and the ileum, in some places, would do little more than admit a good-sized quill. The mesentery more resembled a cake of half an inch thick than anything else; the intestines, mesentery, and omentum, were firmly adherent to each other, and it required a careful dissection to separate them to see their diseased condition. The surface of the peritoneum was covered with little hard bodies, admirably described by Dr. Bright in Guy's Hospital Reports, (vol. v. p. 392, case 30,) as "circular patches of a whitish flesh colour, depressed towards their centre, and scarcely elevated at their circumferences, and marked with radiated vascularity protruding from their centre." These "circular patches," or "tubera," were also found under the pleura costalis.

CASE 2 was one in which a tumor, occupying the left side, and apparently

* London and Edin. Monthly Jour. Med. Science, Dec. 1841.

emerging from under the ribs, was discovered. It was slightly moveable and not very painful to the touch, and had well-defined edges. On dissection it was found that the tumor was only a process of a large medullary mass, lying adherent to the inferior dorsal and superior lumbar vertebræ. It was rather more than six inches in diameter, and flat in form. When cut into, it was found divided into numerous cysts, having a soft centre, and a hard circumference.

CASE 3.—*Disease of the Kidney simulating Disease of the Spleen.*—Robert Small, aged 60, labourer, admitted February 18, 1841. Complains of pain and tenderness over the whole abdomen, but chiefly at the left side, where there is distinct hardness, with well-defined edges. The induration can be traced down to within two inches of the ilium, and forwards to the linea alba. It has the appearance of emerging from under the ribs. On placing the hand on the lumbar region, and pressing forwards, it is felt to move slightly. Left side a little protuberant and dull on percussion; no hardness on the right side, and sound on percussion natural. Both pressure and percussion over the region of the spleen caused pain, but more so over the region of the kidney. Bowels slow: tongue red and dry; pulse 72, pretty firm; urine frequently tested by heat and nitric acid, but not affected by either. It appeared to be natural, both in quantity and quality.

He first began to complain three months before admission, previous to which time he enjoyed good health. The hardness in his side was only observed by him three weeks before he entered the hospital.

There was little variety in the symptoms while he lived. Medicine had no power to relieve his distress. He died two months after admission.

*Inspection* 24 *hours after death.*—Body greatly emaciated; tumor more prominent than during life. The abdomen being opened, the left kidney was found to be greatly enlarged; when taken out it weighed seven pounds imperial. Its structure, when cut into, was medullary, and of a reddish-brown colour. There was not the slightest remains of the natural structure of the kidney; the liver was greatly enlarged, and pervaded by tumors of different sizes, having a medullary structure, and white in colour.

*Case of supposed Sanguineous Extravasation simulating Hernia.*—Thomas Taylor, aged 17, admitted February 20, 1841, affected with a tumor in the left side of the scrotum, of a pyriform shape. It is hard and painful, and can be traced up to the external ring. Four days before admission, while carrying a sack of grain, he felt something give way in the groin, and immediately after perceived the swelling in the scrotum. He applied to a surgeon, who, under the idea that it was a hernia, attempted to reduce it, but failed. He was then sent to the hospital in the state described.

The swelling had neither the elastic feel of an intestinal, nor the doughy one of an omental hernia. As the only inconvenience that the patient suffered was from the pain, the bowels being quite open, no attempts were made after the first to reduce the swelling. Leeches were applied, followed by fomentations. This treatment was several times repeated; the swelling began to subside, the pain had completely ceased, but the tumor was still as hard as ever. Mercurial ointment was now rubbed in, morning and evening, with the best effects: the whole gradually disappeared, and when he left the house, the only remains of the tumor was a small portion about the size of a pea, apparently attached to the cord about its middle. That this was not a hernia, is sufficiently apparent. What seems to be the probable nature of the case is, that a blood-vessel of the cord had burst, and filled the scrotum. The free handling of the parts had excited inflammation, ending in the indurated condition described.

Cases of this sort are always worth reading and recollecting.

### CLINICAL REMARKS ON SOME CUTANEOUS AFFECTIONS. By W. DAVIDSON, M.D., Physician to the Glasgow Infirmary.*

1. *Ioduret of Sulphur for Porrigo.*

Six cases are related. Two will suffice for us.

*Case* 1.—Charles Biggar, aged 10, a vagrant, was admitted on the 1st February 1840. Scattered over the whole of the head were numerous thick greyish patches of scabs, which, when removed, left the surface underneath perfectly bare and shining, but, in a day or two, numerous small pustules made their appearance, accompanied with considerable itching. The eruption appeared in the form of small pustules four years ago. In this case, the scabs were first softened by the constant application of poultices for two days; the head was then shaved. An ointment, composed of five grains of the bichloride of mercury to one ounce of axunge, was tried from the 5th February to the 12th, without any improvement. The following was then employed:—

Iodur. sulphur. - - ℈ij.

Axungiæ - - - ℥ij. Misce.

This ointment was applied daily to the head; and in a few days a decided amendment was remarked.

On the 5th March, the following report was taken.—Pustules and scales are now completely gone, but there are some bald patches on head in the situation of eruption; no itching; surface of skin pretty natural; general health good.

He was dismissed in a few days afterwards.

*Case* 2.—Duncan M'Intyre, aged 10, a singer, admitted 30th December 1840. The whole head, particularly the forehead, was covered with a thick dry greyish white crust, accompanied with itching, but without discharge. The disease was of four years' duration, and was represented to be of a very inveterate kind. General health good; bowels regular; tongue clean; pulse 80.

℞. Iodur. sulphur. - - ℈ij.
Axungiæ - - - - ℥j. Misce.

14*th January* 1841, or a fortnight after the use of the ointment, the eruption is reported to be quite gone, and the skin covering the scalp natural in appearance.

As a precautionary measure, it was continued till the 22d, when he was dismissed.

Dr. Davidson observes:—

"Porrigo, in all its forms, is often a very unmanageable disease, and even when cured is very liable to return. In private practice, and in the hospital, I have tried almost all the external remedies recommended by authors, but have found none so efficacious as the ioduret of sulphur, having repeatedly succeeded in curing the patient permanently with it, after a long trial of other agents. At the same time, it must be remarked, that the evidence derived from the treatment of this disease in an hospital is liable to this objection, that the future history of the case is frequently lost sight of, and there are consequently no means of ascertaining whether or not the disease returns.

The 1st and 4th cases seem to be examples of porrigo scutulata, and both had all the appearances of considerable inveteracy; and I may here remark, that slight cases are rarely sent to this hospital. This species is generally considered the most difficult of cure, and even though the tendency to pustulation be removed,

* London and Edinburgh Journal of Medical Science, No. 12.

there is little dependence to be placed on this sign, until the skin becomes natural in colour, or the hair is beginning to grow.

The 2d, 3d, 5th, and 6th cases seem to be examples of porrigo favosa; the yellow pustules and incrustation, the fetid discharge, the matting of the hair, and the pediculi are all characteristic of this species. It is in general a more manageable disease than the p. scutulata, and is more under the power of the ordinary agents employed in its cure. At the same time, it not unfrequently proves somewhat intractable. In the treatment of porriginous affections, the following is a more particular account than what is given in the short history of the cases. The head is first well washed with soap and water, the hair is then cut as short as possible with scissors, a poultice is applied, and continued for a day or two if necessary, to soften the crusts, which being removed as thoroughly as possible, the hair is closely shaved. In general, the ointment is not applied, until the head has been shaved, but if pediculi be present, it is employed from the commencement, in order speedily to extinguish these vermin. The proportion of ioduret of sulphur employed has varied from 20 to 40 grains to one ounce of axunge; but, in general, the latter quantity may be safely used from the beginning, unless there be some unusual inflammatory action present; for it seldom excites any particular pain or irritation. As a general rule, the daily application of the ointment will be sufficient, but, in some cases, it is advisable to use it twice a-day, in order to facilitate the cure.

Alteratives, or any particular internal treatment, have rarely been resorted to, when the general health was tolerably good. Laxatives have occasionally been prescribed, and a mild farinaceous or milk diet."

We can speak from personal observation for the last two or three years, to the merits of the ioduret of sulphur ointment in the treatment of porrigo. We have found it, on the whole, more serviceable than any other application. In cases of porrigo decalvans we have seen it particularly useful. We employ it of greater strength than Dr. Davidson appears to do. One drachm of the ioduret to seven drachms of lard has been our customary formula, nor have we ever seen that over powerful.

2. *Ioduret of Sulphur for Lepra and Psoriasis.*

*Case.*—Margaret Phillips, aged 11, admitted 14th January 1841. The extremities were covered with circular patches of small shining white scales, which, being detached, exposed a red and somewhat elevated surface. The eruption, which was confined to extremities, made its appearance in the form of small white scales, and had existed for three years. The patient stated, that it has disappeared three or four times, after various remedies had been discontinued. General health was pretty good, tongue clean, bowels regular. Cap. sol. arsenic. gtt. vj. bis in dies.

℞. Iodur. sulphur. gr. xx. Axungiæ, ℥j. Misce.
App. ung. part. affect. omni note.
Hab. baln. calid. secunda quaque nocte.

*25th January.* The strength of the ointment was increased to 40 grains to the ounce of axunge. She was completely cured by 3d February, but remained in the house for eight or ten days longer, expecting her friends from the country to take her home. On the 13th February, she was seized with severe conjunctivitis, accompanied with iritis of right eye, which was subdued in about a fortnight; but the lepra showed no symptoms of return at her dismissal.

*Case.*—John M'Lennan, aged 35, a labourer, was admitted 19th June, 1841. Scattered over trunk and extremities was an eruption, which appeared about eleven weeks ago, in the form of irregular slightly elevated red patches, on the surface of which a thin white scale rapidly formed. Eruption was at first accompanied with slight itching, and the scales being scratched off were rapidly reproduced. Around the knee and ankle joints the patches were more continuous, and the scales considerably thicker than over other parts of body. When scales are removed, the subjacent surface is smooth, dry, and slightly inflamed. He was a patient in ward 10, for a similar affection, about two years ago, when he got well under the use of baths, ointments, &c. General health good.

Hab. baln. tepid. omni nocte.
℞. Iodur. sulphur. ℈ij. Axungiæ ℥j. Misce.
Abrad. cap. Hab. pil. 1. coloc. comp. om. noct.

The ointment was used every night after the bath, and, on the 23d June, the scales were completely removed over whole body, and the prominence of the red patches greatly diminished.

25th.—The amendment was progressing, but a few new papulæ, covered with very thin scales, had appeared on lower extremities, which extended to back and abdomen, accompanied with itching.

Cont. ung. et balneum. Hab. sol. arsen. gtt. x. ter in dies.

*July* 8th.—Eruption almost gone, a few papulæ have appeared on abdomen, general health good.

*July* 12th.—Eruption quite gone, a few reddish stains on skin only remaining.

A case of Lepra Vulgaris is given. It is not remarkably satisfactory.

Dr. Davidson remarks:—

"The ioduret of sulphur does not seem to have so much power over lepra and psoriasis as over porrigo; although, in my experience, it has succeeded more frequently than any other agent that I have tried, with the exception of blistering by cantharides. Lepra alphoides, being a milder disease than lepra vulgaris, is more under its influence, as well as the milder forms of psoriasis, particularly in children; but the species named inveterata would prove somewhat intractable to this as well as to other remedies, as I had occasion to witness in a patient, who, although benefited considerably, was not cured; he was unfortunately carried off by fever before the result was ascertained.

Three other patients affected with lepra and psoriasis have been treated in the Infirmary during last August; two of them have been completely cured, the other is still under treatment, but nearly well; all of them of several years' standing, and having employed a variety of medicines before admission. In one of the cases, a female, who had been using large doses of arsenical solution before her admission, which she was obliged to lay aside, the comparative effects of the ioduret of sulphur, and the acetum cantharidis were tested, the first upon the lower extremities, and the second on the arms. From the result of this and other trials, I am satisfied that the latter is the more powerful as a local agent in this disease. The acet. canth. however, was found too weak when prepared according to the formula in the Edinburgh Pharmacopœia; the proportion of cantharides was therefore doubled. It is proper, however, to state, that strong pyroligneous acid was alone used, without the addition of the acetic. Some other formulæ were tried for the purpose of producing ready vesication, such as an ethereal and an alcoholic solution of cantharides, which succeeded moderately well in some cases; but the speedy evaporation of the menstruum seemed to prevent their thorough action. The following preparation, which was applied with a small brush, answered the purpose pretty well, viz. one part of cantharides to three of a mixture of equal parts of castor oil and alcohol, especially when the flies were suspended in the oil, as suggested by Dr. Leslie, apothecary to the Royal Infirmary. The following liniment, however, which is a modification of the Emp. Canth. E. P., is superior to any preparation that I have tried. It is sufficiently soft during warm weather to be applied with a brush, but requires to be heated when the temperature of the air is low.

℞. Axung. Ol. Rapii, P. Cantharid. ā ℥j.

In order to succeed with either of these vesicating agents, the skin ought to be previously softened either by means of the warm bath, or sponging with warm water."

### 3. *Blistering for Warty Excrescences on the Skin.*

*Case.*—Neil M'Kinnon, plasterer, aged thirty-five, admitted 14th December, 1840. Left lower extremity was twice as thick as right; cellular texture presented the appearance, and gave the sensation of being much hypertrophied, offered much resistance to the fingers when pressure was made, and only pitted slightly. The ham and the two upper and posterior thirds of leg were covered with deep rather fleshy warty excrescences, traversed by deep irregular longitudinal fissures: but in the popliteal space they were transverse. A patch of a similar nature, but dark-coloured, and resembling icthyosis, about the size of the hand, existed over the lower part of the leg at flexion of the ankle joint.

The colour of the patches was generally brownish red. Over anterior part of same leg there were numerous pretty large yellow scabs, and the skin covering the whole of inner and upper part of thigh, was of a dark livid colour, as was also a patch over inner part of the right thigh, which, at one period of the disease, was also encrusted with scabs. The patient ascribed the affection to a fall. It made its appearance in the form of warts on the lower and inner third of the leg about five years ago. He stated that he was cured in this Hospital about two years ago, but that the limb retained its blue appearance, which he says was always the precursor of the warty excrescences. His general health was good, pulse and bowels regular, tongue clean.

App. sol. arsenical. part. affect. quotidie.
Cap. pil. 1 colocynth. comp. omni nocte.

28th *Dec.*—Warty excrescences are in much the same state.

℞. Chlor. zinci ʒj. Aquæ ℥ij. Solve.

App. sol. part. verrucos. Cap. Tk. Canth. gtt. xx. ter in dies.

The strength of the solution of chloride of zinc was gradually increased, until it amounted to three drachms of this salt to an ounce of water, on the 20th January, 1841, while he had been taking the arsenical solution internally from the 11th of the same month; but there was only a slight improvement, there being still an enormous thickness of warty structure. On the 22nd January, a blister was ordered to a portion of the leg, which acted well, and produced a large detachment of warty substance. The blisters were repeated every two or three days until the whole affected surface had been more than once vesicated. Severe strangury was several times produced, even by the tela vesicatoria, which was employed on two or three occasions in this case.

On the 14th February he was dismissed, the right leg appearing to be quite free from warty excrescences, and there remaining on the left only a slight thickening of the skin near the ankle, where the disease resembled icthyosis.

---

### CITY OF DUBLIN HOSPITAL.

### New Method of Reduction for Dislocation of the Hip.*

Two cases are reported.

*Case* 1.—J. M. aged 40, of slight make, and relaxed state of muscular system. The accident happened four hours before admission. The dislocation was on the dorsum of the ilium.

---

* Dub. Med. Press, Sept. 1, 1841.

Considering the relaxed state of the muscular system, it was thought, that this might be a favourable case for trying the method of reduction proposed by M. Colombot. The patient was placed standing and instructed to bend the trunk forwards, so as to support the thorax on a table, the opposite edge of which he grasped with his hands. Mr. Williams now standing on the outer side of the affected limb, bent the leg at a right angle with the thigh, grasping the dorsum of the foot with the right hand, while with the left hand placed at the upper and posterior part of the leg, he exerted a gentle and continued pressure, in which he was aided by the hand of an assistant, placed on his own, and at the same time attempted to dislodge the head of the bone, by directing the thigh somewhat forwards and inwards. After a short time the head of the femur was found to have descended so considerably on the dorsum of the ilium, that it was estimated to be nearly on a level with the acetabulum. The thigh was now suddenly rotated outward, but it was found that the dislocation was not reduced.

This and a second similar attempt having failed, and as from the condition of the muscles, reduction in the ordinary way promised to be attended with little suffering to the patient, the lacs were applied, and extension being made to a very moderate amount, the dislocation was reduced with the utmost facility. Nothing subsequently occurred worthy of notice.

*Case* 2.—This was more successful. The patient, an athletic corn porter, dislocated the femur on the dorsum of the ilium five hours before admission.

Immediately, an attempt was made to reduce the dislocation, the lacs were applied in the usual way, and extension made by three powerful assistants, but it was found impossible to overcome the resistance of the muscles.

About seven hours after the accident, Mr. Williams saw the patient, and resolved to again try M. Colombot's method, which was done precisely as in the preceding case: the mode of extension already described, was persevered in for about three minutes, with the effect of causing a scarcely perceptible change in the situation of the head of the femur on the dorsum of the ilium; the limb was now rotated outwards, as in the preceding case, and the head of the femur re-entered the acetabulum, with the characteristic and in this instance extraordinarily loud report.

The patient experienced scarcely any pain except at the moment when the reduction was effected on rotating the limb outwards. Immediately after the operation the symmetry of the limb was perfectly restored. From the second to the fourth day the dislocated limb was, by measurement, fully three-fourths of an inch longer than the opposite one, but this elongation disappeared by the 6th day, and on the 18th August, the patient, as regarded the accident, was prepared to leave the hospital, but remained to have a small encysted tumor removed from the back of his leg.

The plan is evidently worth a trial.

